# Plant height as an indicator for alpine carbon sequestration and ecosystem response to warming

**DOI:** 10.1038/s41477-024-01705-z

**Published:** 2024-05-16

**Authors:** Quan Quan, Nianpeng He, Ruiyang Zhang, Jinsong Wang, Yiqi Luo, Fangfang Ma, Junxiao Pan, Ruomeng Wang, Congcong Liu, Jiahui Zhang, Yiheng Wang, Bing Song, Zhaolei Li, Qingping Zhou, Guirui Yu, Shuli Niu

**Affiliations:** 1 Key Laboratory of Ecosystem Network Observation and Modelling, Institute of Geographic Sciences and Natural Resources Research, Chinese Academy of Sciences, Beijing, PR China; 2 Department of Environment and Resources, University of Chinese Academy of Sciences, Beijing, PR China; 3 School of Integrative Plant Science, Cornell University, Ithaca, NY USA; 4 Key Laboratory of Sustainable Forest Ecosystem Management-Ministry of Education, Northeast Forestry University, Harbin, PR China; 5 School of Resources and Environmental Engineering, Ludong University, Yantai, PR China; 6 College of Resources and Environment, Southwest University, Chongqing, PR China; 7 Institute of Qinghai-Tibetan Plateau, Southwest Minzu University, Chengdu, PR China; 8 Sichuan Zoige Alpine Wetland Ecosystem National Observation and Research Station, Institute of Geographic Sciences and Natural Resources Research, Chinese Academy of Sciences, Beijing, PR China

**Keywords:** Climate-change ecology, Grassland ecology

## Abstract

Growing evidence indicates that plant community structure and traits have changed under climate warming, especially in cold or high-elevation regions. However, the impact of these warming-induced changes on ecosystem carbon sequestration remains unclear. Using a warming experiment on the high-elevation Qinghai-Tibetan Plateau, we found that warming not only increased plant species height but also altered species composition, collectively resulting in a taller plant community associated with increased net ecosystem productivity (NEP). Along a 1,500 km transect on the Plateau, taller plant community promoted NEP and soil carbon through associated chlorophyll content and other photosynthetic traits at the community level. Overall, plant community height as a dominant trait is associated with species composition and regulates ecosystem C sequestration in the high-elevation biome. This trait-based association provides new insights into predicting the direction, magnitude and sensitivity of ecosystem C fluxes in response to climate warming.

## Abstract (web summary)

Quan et al. show that warming-induced changes in plant community height in a cold, high-elevation region enhance ecosystem carbon sequestration, emphasizing the importance of plant traits in shaping the carbon cycle under climate change.

## Main

There is growing evidence that climate warming is driving shifts in plant species composition with different traits worldwide^1–8^, especially in cold and high-elevation regions^9–13^. However, the ecological consequences of warming-induced changes in plant community structure on ecosystem carbon (C) cycling remain unclear due to the difficulty of mechanistically linking changes in plant community structure to ecosystem function. Plant functional traits directly reflect the ecological strategies of plants and are closely linked to ecosystem functions, regardless of taxonomic differences in species composition. Therefore, the trait approach has been instrumental in advancing our understanding of plant communities’ response to climate change and provides the common mechanistic basis for the role of vegetation in regulating ecosystem C cycling under climate change^14–17^.

In principle, plant species or functional groups with different functional traits respond differently to a certain environmental change. Their diverse responses may lead to shifts in plant community composition and changes in community-level traits under climate change. Consequently, these changes may alter either the direction or the magnitude of trait-related ecosystem C cycling processes in response to climate change. In addition, shifting vegetation composition may also affect the climate sensitivity of ecosystem C fluxes, which quantifies the intensity of the ecosystem C–climate feedback^18–20^. However, our poor understanding of the underlying mechanisms has led to large uncertainties in predicting ecosystem stability and C budgets under ongoing climate change. The trait approach may provide new insights into this issue, but currently, there is a dearth of studies investigating the relationships between plant community traits and the climate sensitivity of ecosystem C fluxes.

Plant height may be one of the most important traits determining the response of plant community composition to climate warming, particularly in cold regions^21–23^. In temperature-limited ecosystems, warming can relieve temperature limitation and lead to increased plant growth and plant height. Consequently, at the community level, increased plant height intensifies aboveground light competition. Taller species shade and exclude shorter ones^24^, leading to shifts in plant community composition and an overall increase in community height. Synthesis of warming experiments and long-term monitoring conducted across various spatial and temporal scales have revealed that the increase in plant community height is attributed to shifts in community composition in response to climate warming^23,25,26^. For example, ref. ^22^ synthesized 61 warming studies ranging from 1 to 20 years across high-latitude tundra ecosystems worldwide and found significant increases in plant community height in response to warming. They attributed this pattern to warming-induced canopy closure and the resulting increased light competition that favoured taller, vertically growing species. Also, by exploring 7 key traits from 117 sites over 27 years across the cold tundra biome, ref. ^21^ showed that when weighted to the community level, plant height was the only trait that increased with rising temperature, mainly due to turnover of taller species. In particular, height is considered to be a crucial trait for species’ C acquisition strategy due to its dominant role in light competition and its strong correlations with numerous traits for C cycling^27–31^. These studies, along with others, have suggested that ecosystem functions related to plant height, especially C cycling processes, will change rapidly in response to climate change^27,32,33^. However, the lack of empirical evidence leaves a significant gap in our understanding of how warming-induced changes in plant community composition and height can affect the direction, magnitude and sensitivity of ecosystem C cycling in response to climate warming.

In this study, we combined an in situ manipulative warming experiment with a field survey along a 1,500 km transect across the Qinghai-Tibet Plateau (QTP) to investigate both the general pattern and the underlying mechanism of how warming-induced changes in plant community composition and height alter ecosystem C sequestration in this high-altitude, cold region. Linking plant community structure to ecosystem function under climate change remains a challenge in assessing the global change impact. Here we established a link between climate warming, plant community structure and traits, and net ecosystem productivity (NEP, the difference between the CO_2_ absorbed by vegetation through photosynthesis and the CO_2_ released through ecosystem respiration). This integration of multiple processes provided a unique understanding of the complex response and regulation of the ecosystem to climate warming. Specifically, we aimed to address the following questions: (1) How does warming change plant community composition and height? (2) Do these changes accelerate or counteract the direct warming impact on NEP? and (3) How will changes in plant community height under warming impact soil carbon content?

## Results

### Changes in the plant community in the warming experiment

**Fig. 1:**
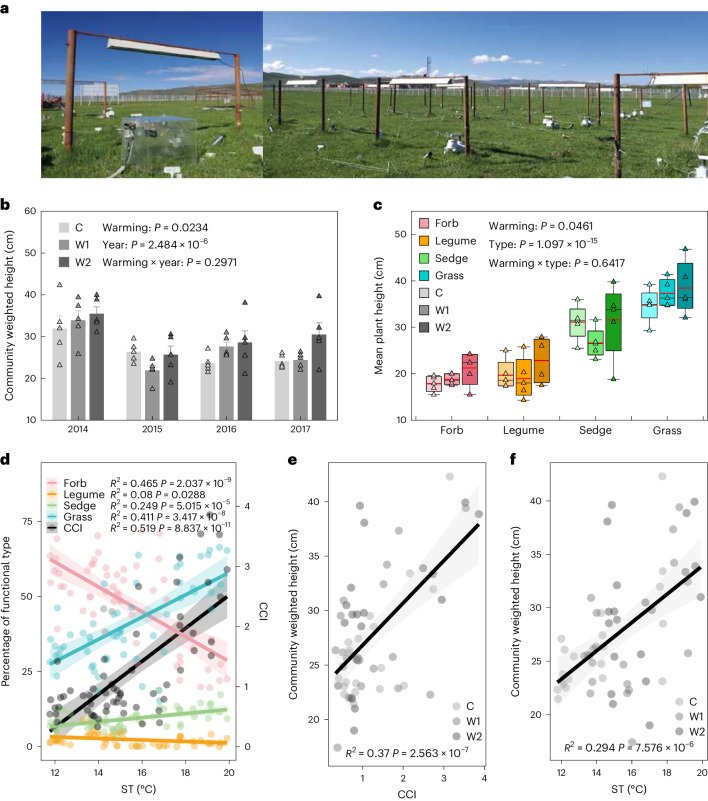
Changes in plant community weighted height and composition under warming. **a**, Photographs by authors show the field warming experimental plots at the study site. **b**, Plant community weighted height under control (C), low-level warming (W1) and high-level warming (W2). Data are presented as mean ± s.e.m.; *n* = 5. **c**, Mean plant height of the four functional types under different warming treatments (*n* = 5). In boxplots, the lower and upper boundaries show the 25th and 75th quartiles, the black lines inside the boxes indicate the median, the red lines represent the means and the whiskers represent 1.5 times the interquartile range. **d**, Relationships between mean ST during the growing season and the proportion of the ANPP of grass, forbs, legumes and sedges in the community (left *y* axis) and the CCI (right *y* axis) across years and plots (*n* = 60). **e**,**f**, Relationships between community weighted height and CCI (**e**) (*n* = 60) and ST (**f**) (*n* = 60) in the warming experiment. In **b** and **c**, repeated-measures ANOVA and two-way ANOVA with two-sided test were respectively used to determine significant effects. Significance of the effects are indicated by exact *P* values. In **d**–**f**, linear regression with two-sided test was used for the statistical analysis, and the coefficients of determination (*R*^2^) and the exact *P* values are indicated. The error bands are 95% confidence intervals (CIs) (±1.96 s.e.m.) around the fitted regression lines.

Across the experimental period, low and high-level warming increased mean soil temperature (ST) during the growing season by 1.41 °C and 2.44 °C on average, respectively. Overall, warming significantly increased plant community weighted height (Fig. 1b). On the one hand, warming had positive effects on the mean plant height of different functional types (Fig. 1c). On the other hand, warming promoted taller functional plants within the community and altered plant community composition. The proportion of grass in the community ranged from 15.29% to 66.35%, and the proportion of forbs ranged from 19.59% to 72.01% (Fig. 1d). The changes in these four plant functional groups were closely related to changes in ST. Across all years and plots, the proportion of grass and sedge in the community significantly increased with increased ST, while the proportion of forbs and legumes significantly decreased with increased ST. Thus, the ratio of grass and sedges to forbs and legumes, indicated by community composition index (CCI), significantly increased with ST (Fig. 1d). Furthermore, the grass and sedges were significantly taller than the legumes and forbs (Fig. 1c and Supplementary Table 1). The mean plant heights (±s.d.) of the four functional types were 36.82 ± 8.52 cm, 29.76 ± 7.82 cm, 20.43 ± 6.10 cm and 19.28 ± 3.72 cm for grass, sedges, legumes and forbs, respectively. Overall, changes in CCI led to increased plant community weighted height in response to warming (Fig. 1e,f).

### C uptake responds to plant height in the warming experiment

**Fig. 2:**
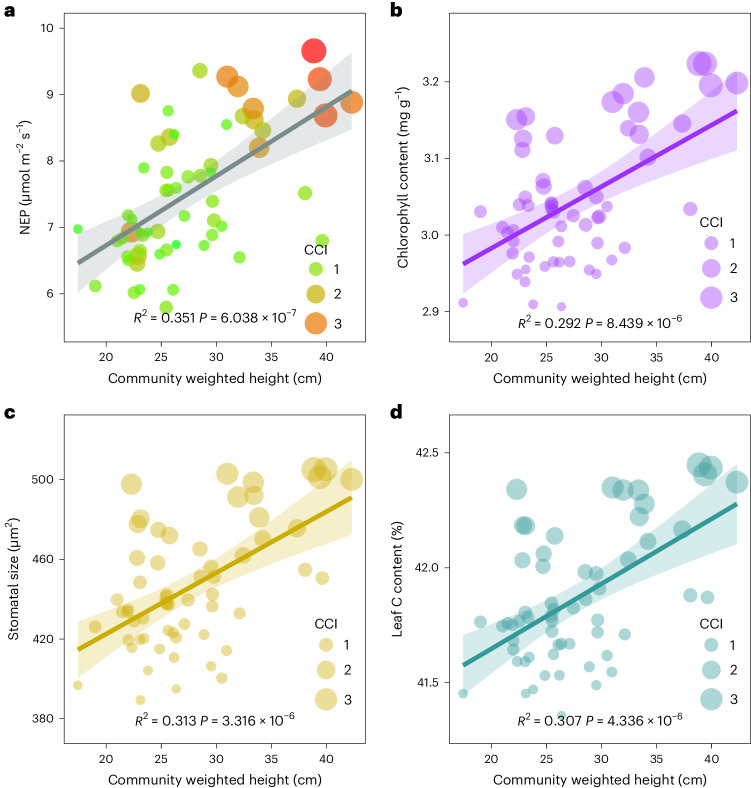
Relationships of plant community weighted height with NEP and plant functional traits in the warming experiment. **a**–**d**, Relationships of plant community weighted height with NEP (**a**) (*n* = 60) and with community weighted plant functional traits for chlorophyll content (**b**) (*n* = 60), stomatal size (**c**) (*n* = 60) and leaf C content (**d**) (*n* = 60) in the warming experiment. The point size indicates the CCI. Linear regression with two-sided test was used for the statistical analysis, and the coefficients of determination (*R*^2^) and the exact *P* values are indicated. The error bands are 95% CIs (±1.96 s.e.m.) around the fitted regression lines.

Changes in plant community height could impact the uptake of ecosystem CO_2_ as plant height reflects its light capture capacity and C use strategy. We found a significant positive relationship between NEP and community weighted height accompanied by changing CCI (Fig. 2a). Since changes in community weighted height can be attributed to both height variability and changes in community composition, we disentangled them and evaluated their effects on NEP. We found that both changes in height and community composition were positively correlated with NEP (Supplementary Fig. 1). Furthermore, to explore the mechanisms underlying the increased NEP with a community shift towards taller plants, we examined plant functional traits and found that taller species had higher chlorophyll content and larger stomata (Supplementary Methods and Fig. 2), which may have benefited community photosynthesis. In the controlled warming experiment, we did not consider the potential impacts of warming treatment on the intraspecific variation of plant traits such as chlorophyll content, stomatal size and leaf C content. Nevertheless, at the community level, taller plant communities were characterized by higher values of these traits (Fig. 2 and Supplementary Methods), which were associated with higher NEP (Supplementary Fig. 3).

**Fig. 3:**
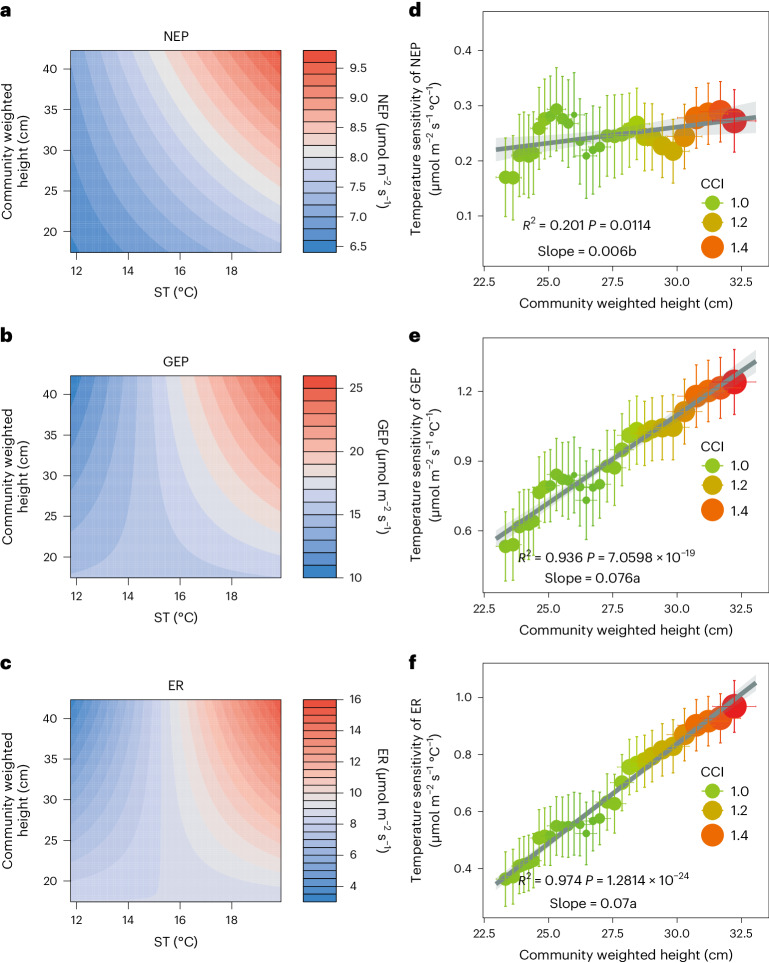
Partial relationships between mean ST during the growing season, community weighted height, ecosystem C fluxes and their temperature sensitivities in the warming experiment. **a**–**c**, Surface plots showing the partial relationships between mean ST during the growing season, community weighted height and NEP (**a**) (*n* = 60), GEP (**b**) (*n* = 60) and ER (**c**) (*n* = 60) in the warming experiment. **d**–**f**, Temperature sensitivities of NEP (**d**) (*n* = 60), GEP (**e**) (*n* = 60) and ER (**f**) (*n* = 60) change with community weighted height. The temperature sensitivities are the slopes of rolling regressions between ST and ecosystem C fluxes along the community weighted height gradient with rolling width of 30. In **d**–**f**, data are presented as mean ± s.e.m., with point size indicating CCI. Linear regression with two-sided test was used for the statistical analysis. The fit statistics (slopes, *R*^2^ and exact *P* values) are provided. The error bands are 95% CIs (±1.96 s.e.m.) around the fitted regression lines. Different letters next to the slopes indicate significant differences among slopes.

Surface plots showing the partial relationships between community weighted height, ST and ecosystem C fluxes indicated that the temperature sensitivity of NEP and its two components were largely modified by plant community height. Under lower community height range, increasing ST rarely increased NEP and its two components, but under a higher height range, NEP and its two components had a more pronounced increase with increased ST (Fig. 3a–c). Results from rolling regression analysis verified this pattern, which showed that the temperature sensitivities (the regression slopes of ST–C fluxes) of NEP, gross ecosystem productivity (GEP) and ecosystem respiration (ER) significantly increased with community weighted height (Fig. 3d–f and Supplementary Fig. 4). Thus, the NEP, GEP and ER of taller plant communities had higher temperature sensitivities.

### C uptake responds to plant height in the transect study

**Fig. 4:**
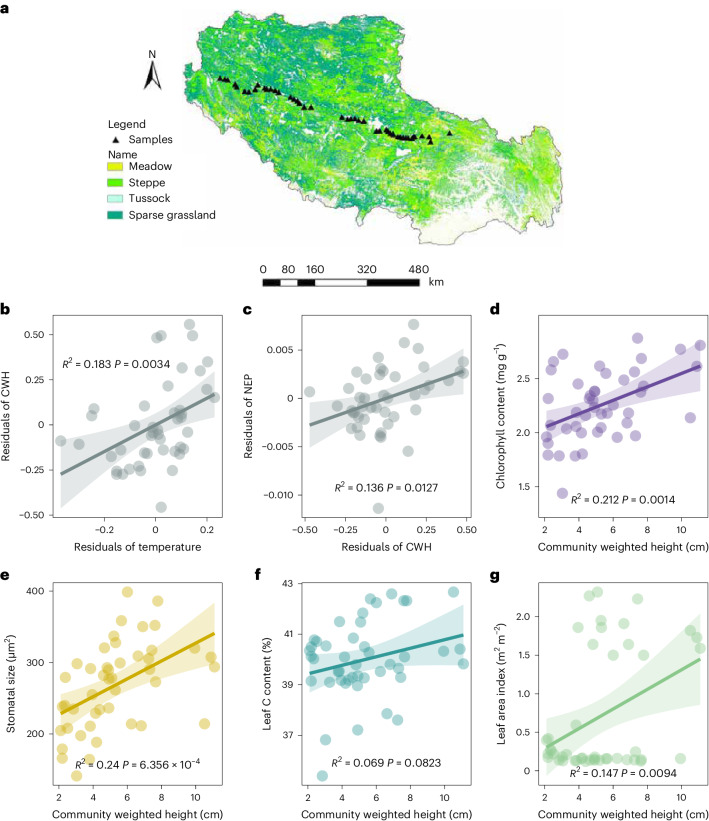
Partial relationships of CWH with temperature and NEP, and the relationships of CWH with plant functional traits in the transect study. **a**, The spatial distribution of investigation sites. **b**, Partial relationship of CWH with mean annual temperature after controlling for the effects of mean annual precipitation, soil total nitrogen, available phosphorus and study site in the transect study (*n* = 45). **c**, Partial relationship of NEP with CWH after controlling for the effects of mean annual temperature, precipitation, soil total nitrogen, available phosphorus and study site in the transect study (*n* = 45). **d**–**g**, Relationships of CWH with community weighted plant functional traits for chlorophyll content (**d**) (*n* = 45), stomatal size (**e**) (*n* = 45), leaf C content (**f**) (*n* = 45) and leaf area index (**g**) (*n* = 45) in the transect study. In **b** and **c**, a linear mixed-effect model was used to estimate the partial relationships. Partial regression with two-sided test was used. In **d**–**g**, linear regression with two-sided test was used for the statistical analysis. The coefficients of determination (*R*^2^) and the exact *P* values for all the regressions are indicated. The error bands are 95% CIs (±1.96 s.e.m.) around the fitted regression lines.

We found that changes in plant community composition and the consequent increase in community height promoted ecosystem C uptake in the field warming experiment. To test the scalability of this mechanism in influencing ecosystem C uptake at the regional scale, we conducted a field investigation at 45 sites along a 1,500 km transect distributed across the QTP. Overall, in the transect study, we found a similar pattern where plant communities tended to be taller at warmer sites, even after controlling for the effects of precipitation and soil nutrient status, such as soil total nitrogen and available phosphorus (Fig. 4b). Furthermore, despite removing the confounding effects of temperature, precipitation and soil nutrient status, we still found that NEP (negative value of net ecosystem CO_2_ exchange, derived directly from the remote sensing data without additional validation) increased with plant community height (Fig. 4c and Supplementary Methods) in this high-altitude and cold region. Similarly, we also found that across the investigation sites, plant community height was positively correlated with chlorophyll content, stomatal size, leaf C content and leaf area index (LAI) at the community level (Fig. 4), which also increased NEP (Supplementary Fig. 5). Furthermore, the soil C content increased with plant community height and NEP in this region (Supplementary Fig. 6).

## Discussion

Our warming experiment provides direct evidence that shifts in plant communities towards a taller height under warming could promote ecosystem C uptake in alpine ecosystems (Figs. 2 and 3, and Supplementary Fig. 7). The field investigation along a 1,500 km transect across the QTP region supported the generality of this result by showing that the taller plant community height was associated with higher NEP and soil total C content (Fig. 4 and Supplementary Fig. 6). Taken together, both evidence from the warming experiment and the large-scale field investigation suggested that a taller plant community height is indicative of enhanced ecosystem C sequestration.

This promotion of ecosystem C uptake was probably due to the higher C uptake capacity of taller plant communities and the strong positive correlations between plant height and traits for light capture and C uptake such as LAI or leaf mass per unit area^27,28,31,32^. Results from our warming experiment supported this by showing that the increased proportion of taller functional species, such as grass and sedges, in the community significantly enhanced community height and NEP, while the increased proportion of shorter forbs in community significantly reduced community height and NEP (Supplementary Fig. 7). Thus, increased community height with changing community composition under warming significantly enhanced NEP (Fig. 2). Moreover, taller plant species have higher chlorophyll content and larger stomatal size (Supplementary Methods and Fig. 2), indicating higher C uptake capacity of taller plant species. We found consistent positive relationships between plant community height and chlorophyll content, stomatal size and leaf C content at the community level in both warming experiment (Fig. 2) and regional investigation (Fig. 4). It implies that taller plant communities could potentially sequester more C into the ecosystem because of changes in C cycling-related community traits associated with increasing community height. This was supported by the positive relationships between NEP and these community traits in both the experimental and transect studies (Supplementary Figs. 3 and 5). Therefore, we expected that the increased C uptake in response to increasing community height under warming would promote ecosystem soil C sequestration in the long term. Accordingly, even not reaching the significance level (*P* > 0.05) during our experimental period, overall, we found that soil total C content increased by 0.25% under high-level warming during the experimental years (Supplementary Fig. 8). This increasing trend of soil C content was also confirmed by a modelling study in this region^34^ and by our long-term warming experiment^35^ and transect investigation (Supplementary Fig. 6).

Theoretically, various processes govern soil carbon sequestration under warming. Warming can increase root production^36–38^ and exudates^39,40^, enhancing plant-derived soil C input^41^. It also stimulates microbial turnover, residues and C use efficiency^42–44^, increasing microbial-derived soil C^45,46^. Conversely, warming will promote soil C loss by increasing microbial respiration^47,48^ and enhancing enzyme activities^49,50^. These possible mechanisms make it difficult to understand soil C changes under warming, which requires future investigation. Here, our study provides a new insight into projecting ecosystem C sequestration under climate change from a plant trait perspective by revealing the potential association between soil C and plant community traits. Overall, both the warming experiment and the field investigations showed enhanced ecosystem C uptake associated with increased community height under warming.

It is also supposed that the enhancement in ecosystem C uptake in response to increased plant community height may be due to the change in plant biomass, since studies have suggested that plant primary production could affect both ecosystem C uptake and release^51,52^. In this study, we did not detect significant warming effects on aboveground net primary production (ANPP) in either the warming experiment (*P* = 0.061) or the transect study (*P* = 0.423). This may be attributed to the compensatory effects among different plant functional groups competing for light under warming (Fig. 1c,d and Supplementary Fig. 13). Moreover, after controlling for the effect of ANPP, we still found positive relationship between community weighted height and NEP and soil total C (Supplementary Figs. 9 and 10). In particular, we found that mass-specific C uptake rates increased with community height (Supplementary Fig. 11) in both the warming experiment and the transect study, which implies that taller plant communities can fix C more efficiently. Therefore, our study highlighted that plant community height could be an underlying trait-based mechanism that is responsible for the impact of warming on ecosystem C sequestration. The mechanistic understanding of the plant trait-based linkage between ecosystem structure and function under climate warming will improve our confidence in predicting ecosystem C balance.

Both an increase in the mean height of different functional groups and a shift in community composition towards taller plant species contributed to increased plant community height in the warming experiment. Taller grass and sedge species were favoured by higher temperatures than shorter forbs and legumes (Fig. 1). Similarly, positive warming effects on grass^11,53,54^ and sedges^13^ and negative warming effects on legumes^53^ and forbs^11,54^ have also been found previously. Existing studies suggested that warming-induced water limitation would suppress shallow-rooted plants and favour deep-rooted plants, resulting in plant functional changes in those dry ecosystems^11,13,54,55^. However, this mechanism may not be the main driver of changes in community composition in ecosystems where water availability is not limiting. A precipitation exclusion experiment adjacent to our experimental site showed that plant community structure did not change under rainfall reduction until extreme drought condition was reached (that is, one twelfth of the ambient rainfall)^56,57^. We also found a significant partial relationship between CCI and temperature instead of annual rainfall (Supplementary Fig. 12) in the warming experiment. These results indicate that rising temperature plays a dominant role in altering plant community composition at our warming experimental site where water is typically not limited.

Height is identified as a key trait for plant ecological strategy, especially for light competition^27,29–31,33^. The competitive advantage of taller species for light may serve as the underpinning mechanism for species turnover or species loss in a community, especially under fertilization or warming^21,22,27,30,33^. By relieving temperature limitation in cold ecosystems, warming may promote plant growth and light competition. Taller plants can position their leaves in the upper canopy and thus have a competitive advantage for light interception over shorter plants^29,30^, which can lead to changes in plant community composition and structure. In our warming experiment, we found a dramatic decline in light intensity from the top of the canopy to the ground surface, which indicated a strong shading effect of taller plants (Supplementary Fig. 13). Moreover, the observed increase in grasses and sedges or decrease in forbs and legumes in our warming experiment was in accordance with their height, which contributed to taller plant community height. Our findings suggest that plant height may be a common mechanism in determining the response of species turnover to climate change.

This work advances our understanding of trait-based regulation of ecosystem C sequestration in response to climate warming in the following aspects. First, the direct impact of warming by changing temperature and the indirect impact of warming by changing plant community composition on ecosystem C exchange have not been explicitly disentangled or evaluated in previous studies. The observed changes in ecosystem C flux in response to warming are actually the integration of outcomes from both the above biotic and abiotic impact pathways. They are governed by distinct mechanisms. By separating their effects in our warming experiment, the partial relationships showed no significant difference between the slopes of ST and CCI for NEP, which indicated that plant community change could exert a comparable impact on net ecosystem C flux to that of temperature change (Supplementary Fig. 14). This change in plant community structure under warming would accelerate the direct positive effect of increasing temperature on ecosystem CO_2_ uptake. Particularly, changes in plant community traits can directly explain the community change effect on ecosystem C fluxes under warming, so we propose that the dynamics of plant community traits concurrent with community structure under warming should be taken into account when modelling and predicting the response of ecosystem C balance to climate change. This consideration is especially important in cold or high-elevation regions where plant communities are sensitive to climate change^9,11–13,21^.

Second, our work provides a unique insight into the changes in temperature sensitivity of ecosystem C fluxes from a trait-based perspective. Our current limited understanding of the temperature sensitivity of ecosystem C flux has led to a large uncertainty in assessments of ecosystem C balance and projecting reliable future climate scenarios^58,59^. The oversimplification of using a constant temperature sensitivity value by many earth system models can cause inaccuracy and discrepancy in modelling ecosystem C flux and budget^59–61^. Although a few previous studies have found that changes in vegetation composition are responsible for altered temperature sensitivity of ecosystem C fluxes, the underlying mechanisms remain obscure^18,62,63^. Our findings showed that plant community traits could be the underlying mechanisms. In our warming experiment, we found that both GEP and ER became more sensitive to rising temperature with increasing community height (Fig. 3 and Supplementary Fig. 4). However, the relative trade-off between GEP and ER did not change since we did not detect significant differences between their slopes (Fig. 3). Similar results were also found by a precipitation exclusion experiment and a nitrogen addition experiment adjacent to our study site, which suggested that the trade-off between GEP and ER also remained stable under changing water^64^ and nutrient^65^ conditions in this region. Moreover, we found that the temperature sensitivity of NEP increased with increasing plant community height, which implies that for the same temperature rise, ecosystems with a taller plant community would sequester more CO_2_ from the atmosphere. Our study adds a unique, trait-based insight into shifts in plant community structure and traits, and their association with the temperature sensitivity of ecosystem C fluxes. Thus, we suggest that more attention should be paid to the effect of plant traits on the temperature sensitivity of ecosystem C fluxes. The incorporation of vegetation and temperature sensitivity dynamics into models is essential for projecting ecosystem C fluxes under future warming scenarios^66,67^.

Third, earth system models are trying to incorporate key plant traits to improve projection of ecosystem change^16,68,69^, since plant traits are measurable characteristics that not only determine the response of the plant community to climate change but also drive changes in ecosystem functions^70^. Our study informs model projection of ecosystem C budget in cold, high-elevation ecosystems under climate warming by highlighting the crucial role of plant community height. We acknowledge that our research combined a transect study to identify the general patterns with a controlled warming experiment to explore the underlying mechanisms of changing plant community structure and traits, and their impacts on ecosystem C cycling under warming. However, some caution is required. Warming treatment can enhance NEP by stimulating plant physiological activity and photosynthesis^71,72^, lengthening the growing season^73^ or improving nutrient availability^74^. In addition, potential confounding effects, such as water and nutrient status, may bias the response of NEP to plant community height in the transect study. Nevertheless, after removing these confounding effects by partial regression, we still found a positive relationship between plant community height and NEP (Fig. 4b,c and Supplementary Tables 3 and 4). Particularly, plant community height increased with rising temperature, regardless of confounding water and nutrient status (Fig. 4b and Supplementary Fig. 15a). After accounting for the covariation among the influencing factors, the structural equation models also supported these findings. The results revealed that, in this temperature-limited region, community height served as the key plant trait through which warming impacted NEP (Supplementary Fig. 16). Moreover, after controlling for the collinearity among the potential covariates, the ridge regression results further supported the critical role of plant community height in driving the variation in NEP (Supplementary Fig. 17). These patterns remained consistent in both the controlled warming experiment and the transect study. The findings confirmed that a warmer climate is more favourable for taller plant communities in cold regions, which could benefit ecosystem C uptake. Overall, our work provides evidence linking climate change, plant community composition, plant functional traits and ecosystem function together. The success of this attempt suggests that further efforts are needed to identify the key traits that determine ecosystem function and its response to climate change.

## Conclusion

By combining a manipulative warming experiment with a regional field investigation across the high-altitude QTP, our study shows that warming could promote plant community height, which would benefit ecosystem C sequestration in this cold region. We also found that the shift in plant structure with taller community height could increase the temperature sensitivity of ecosystem C uptake, implying that for the same temperature increase, ecosystems with higher plant communities would fix more C from the atmosphere. We highlight that an increase in plant height at the community level is crucial in shaping the ecosystem C budget under climate warming. This study provides a new insight into the projection of C balance of ecosystems experiencing both climate change and shifts in plant community composition and traits. Our findings suggest that modelling efforts should account for vegetation dynamics and associated changes in thermophilic traits when predicting ecosystem change and feedback in the future.

## Methods

### Warming experiment

The warming experiment was conducted in an alpine meadow on the Eastern Qinghai-Tibetan Plateau (32° 48′ N, 102° 33′ E), which is in Hongyuan County, Sichuan, China, at an altitude of ~3,500 m. Over the past 60 years, the mean annual precipitation has been 753 mm, ~80% of which occurs between May and September. The mean annual temperature is 1.1 °C with monthly temperature ranging from −10.3 °C in January to 10.9 °C in July. The soil at the study site is classified as Cryumbrept following US Soil Taxonomy^75^. The plant species in this alpine meadow are dominated by *Deschampsia caespitosa* (Linn.) Beauv., *Kobresia setchwanensis* Hand.-Mazz., *Carex schneideri* Nelmes and *Anemone rivularis* Buch.-Ham^76^.

A randomized complete block design with three warming treatments and five replications was used in this study. Three 3 × 2 m plots laid out in each of five blocks were randomly assigned to three treatments: control, low-level warming (+1.5 °C) and high-level warming (+2.5 °C). The warming plots were heated continuously since June 2014 using 165 cm (length) × 15 cm (width) infrared radiators (MSR-2420, Kalglo Electronics) suspended in the centre, 1.5 m above the ground. The heaters for the low-level warming treatments were set at a radiation output of 1,000 W, while the heaters for the high-level warming treatments were set at a radiation output of 2,000 W. In each control plot, a dummy heater with the same size and shape as the infrared radiator was suspended at the same height to simulate the shading effect. The adjacent two plots were 3 m apart.

Ecosystem C fluxes were measured twice per month from June 2014 to September 2017 over the growing season (from May to September). In May 2014, a 0.5 × 0.5 m square aluminum frame was inserted into the soil to a depth of 3 cm in each plot with a distance of at least 30 cm to the plot’s border. Care was taken to minimize soil disturbance during installation. Frames provided a plane base between the canopy chamber (0.5 × 0.5 × 0.5 m, polymethyl methacrylate) and the soil surface. Ecosystem CO_2_ fluxes were measured with an infrared gas analyser (LI-6400XT, LI-COR) attached to the transparent canopy chamber. During measurements, the chamber was sealed to the soil surface by attaching it to the permanently fixed aluminum base in each plot. Two small fans ran continuously to mix the air inside the chamber during the measurement. We took consecutive recordings of CO_2_ concentration once every 10 s in 80 s after steady-state conditions were achieved in the chamber. The flux rates were determined from the time course of the concentration to calculate the NEP. Following the NEP measurements, the chamber was vented to exchange air with the outside, then replaced on the aluminum frame and covered with an opaque cloth to measure ecosystem respiration (ER). Gross ecosystem productivity (GEP) was the sum of NEP and ER, calculated as in equation (1).

1 $${\rm{GEP}}={\rm{NEP}}+{\rm{ER}}$$

All measurements were performed simultaneously under cloud-free conditions. Soil temperature at a depth of 10 cm was measured using a thermocouple probe connected to the Li-6400XT at the time of ecosystem CO_2_ fluxes measurement. Annual precipitation was collected from the nearby meteorological station around the study area.

To investigate the plant community composition, ANPP was measured by clipping all living plants at the ground level in a 0.1 × 1 m quadrat in each plot in the middle of August every year from 2014 to 2017, when biomass peaked. All plants were sorted to species and oven dried at 65 °C for 48 h and weighed.

The height of each species was measured in each plot in a permanent 0.5 × 0.5 m quadrat, which was at least 30 cm away from the plot edges. Each quadrat was evenly divided into 25 subquadrats, and the plant height of one healthy individual of each species that occurred in each subquadrat was measured and recorded, then, plant species height was obtained by averaging across the 25 subquadrats for each species.

### Regional field investigation

A field investigation of 45 study sites along a 1,500 km transect in the QTP grasslands was conducted during the peak growing period between July and August in 2019. The lowest mean annual temperature among all sites was −3.5 °C and the highest was 1.8 °C. The lowest mean annual precipitation was 71.9 mm and the highest was 461.5 mm. The average elevation was 4,576 m (Supplementary Table 2). The dominant species of these study sites were *Kobresia pygmaea* C. B. Clarke, *Stipa purpurea* Griseb, *Astragalus polycladus* Bureau et Franch, *Leontopodium hayachinense* Hara et Kitam and *Aster hispidus* Thunb.

At each site, ten 0.5 × 0.5 m quadrats were randomly selected to measure plant community composition, ANPP and plant species height. The methods we used were the same as those used in the warming experiment. Soil samples were randomly collected from 3 of the 10 quadrats by using a 7.5-cm-diameter soil auger at a depth of 10 cm. Then, soil samples were air dried and sieved with a 2 mm mesh to remove stones and plant roots. A LECO macro-CN analyser (LECO) was used to measure soil total C and total nitrogen. Soil available phosphorus was measured using the molybdenum-antimony anti-spectrophotometric method. Mean annual temperature and precipitation (1987–2017) of these investigation sites were obtained from the Loess Plateau Scientific Data Center (http://loess.geodata.cn/) with Kriging interpolation.

### Statistical analysis

Previous knowledge suggested that the warming effects may differ among different plant functional types such as grass^53,54^, forbs^11,54,77^ and sedges^13^. We classified all plants into different functional types, including grass, sedges, legumes and forbs. We found positive effects of increasing temperature on grass and sedges, which contrasted with effects on legumes and forbs (Fig. 1d). On the basis of their positive or negative responses, we classified the four plant functional types into two groups, and employed the CCI to identify and quantify the directional changes in plant community composition. The CCI was calculated as:

2 $${\rm{CCI}}=\left({{P}}_{{\rm{grass}}}+{{P}}_{{\rm{sedge}}}\right)/\left({{P}}_{{\rm{legume}}}+{{P}}_{{\rm{forb}}}\right)$$

where *P*_grass_, *P*_sedge_, *P*_legume_ and *P*_forb_ represent the proportion of ANPP of grass, sedges, legumes and forbs in the community, respectively. Overall, the CCI increased significantly in response to increasing temperature (Fig. 1d), implying a directional change in plant community composition and that the CCI index can quantify the compositional changes in plant community composition.

Mean plant height of each functional group was the average of plant heights of all species within each functional group. On the basis of the mean plant height of each functional group and the percent ANPP of each functional group in the community, we calculated the CWH as:

3 $${\rm{CWH}}=\mathop{\sum }\limits_{i}^{S}{p}_{i}\times {H}_{i}$$

where *p*_*i*_ is the percent ANPP of functional group *i*, *H*_*i*_ is the mean plant height of functional group *i* and *S* is the number of functional groups in the community.

Two-way analysis of variance (ANOVA) was used to explore the effects of warming treatment, plant functional type and their interaction on mean plant height. Repeated-measures ANOVAs were performed to examine the effects of warming treatment, year, plant functional group and their interactions on community weighted height. Warming treatment, year and plant functional type were set as fixed effects and the block was set as a random effect. A surface plot was applied using the ‘visreg (v.2.7.0)’ package in R v.3.4.3 to visualize the partial relationships and interactive effects of ST and community weighted height on ecosystem C fluxes^78,79^. To further explore how the temperature sensitivities of ecosystem C fluxes changed with community weighted height, rolling regression was used to analyse the changing relationships (slopes) between ST and ecosystem C fluxes along increased community weighted height. Rolling regression estimated slopes between ST and ecosystem C fluxes using a fixed rolling width (numbers of raw data) of subsets of the full data set along increased community weighted height. The rolling regression was applied using the ‘zoo (v.1.8-12)’ package.

We used a linear mixed-effect model to explore the effects of environmental variables on CWH. In the controlled warming experiment, ST and mean annual precipitation were set as fixed effects and the block was set as a random effect. In the transect study, mean annual temperature, precipitation, soil total nitrogen and available phosphorus were set as fixed effects and study site was set as a random effect. In addition, in both the controlled warming experiment and the transect study, CWH and those environmental variables were included as fixed effects in the linear mixed-effect model to examine their effects on NEP. The models were fitted using restricted maximum likelihood estimation with the ‘nlme (v.3.1-163)’ package. On the basis of the models, partial residuals were used to explore the specific partial relationships: (1) between CWH and ST, controlling for the effects of mean annual precipitation and block in the controlled warming experiment; (2) between CWH and mean annual temperature, controlling for the effects of mean annual precipitation, soil total nitrogen, available phosphorus and study site in the transect investigation; (3) between NEP and CWH, controlling for the effects of ST, mean annual precipitation and block in the controlled warming experiment; and (4) between NEP and CWH, controlling for the effects of mean annual temperature, precipitation, soil total nitrogen, available phosphorus and study site in the transect investigation. Partial regressions were also used to test the specific partial relationships between the response variables and the predicting variables. Analysis of covariance was used to test the differences among slopes.

### Reporting summary

Further information on research design is available in the Nature Portfolio Reporting Summary linked to this article.

### Supplementary information

Supplementary Information Supplementary Methods, Tables 1–4 and Figs. 1–17.

Reporting Summary

## Data Availability

All data reported in this paper have been deposited in figshare (10.6084/m9.figshare.23519208)^80^.
