# Supplementary material for: Plant height as an indicator for alpine carbon sequestration and ecosystem response to warming
Source: Nat Plants. 2024 May 16;10(6):890–900. doi: 10.1038/s41477-024-01705-z (PMC11208140; doi:10.1038/s41477-024-01705-z)
Supplement: Supplementary file 1 — Supplementary Methods, Tables 1–4 and Figs. 1–17. [file 41477_2024_1705_MOESM1_ESM.pdf]

nature plants

Article https://doi.org/10.1038/s41477-024-01705-z

Plant height as an indicator for alpine carbon sequestration and ecosystem response to warming

In the format provided by the authors and unedited

Supplementary Methods

Disentangling height variability and community composition effects

Because variation of community-weighted trait can be caused by both trait variability and changes in the community composition, we disentangled community-weighted height (CWH) into both height variability and changes in community composition following Leps et al. (2011) [81]:

$$HV = CWH - CWH_{fixed} \tag{1}$$

where CWH is community weighted height defined in Methods section in the main text. CWH_{fixed} is 'fixed' community weighted height caused by changes in community composition alone. Specifically, it was calculated using 'fixed' mean height of each plant functional group across all communities and weighted by plot-specific functional composition data. As the difference between them, HV is the height variability which represents changes in height alone. By disentangling their roles, we can evaluate the effects of height variability and community composition on net ecosystem productivity, which are shown in Supplementary Figure 1.

Soil C content measurement in warming experiment

Soil samples were randomly collected from each plot once a year from 2015 by using a soil auger (7.5 cm in diameter, 10 cm in depth). All samples were air-dried and sieved with 2 mm mesh to remove stones and plant roots. We used a LECO macro-CN analyzer (LECO, St. Joseph, MI, USA) to measure soil total C. Changes in soil total C content under three warming treatments are shown in Supplementary Figure 8.

Light intensity measurement in warming experiment

We measured light intensity by using a digital light meter (TES-1335, TES Electrical Electronic Corp., Taiwan, China) at the control plots in August 2021 when biomass peaked. The light intensities at five heights from ground surface to canopy (0, 10, 20, 30, 40 and 50 cm) of plant community were measured under cloud-free condition. The differences in light intensity among different heights are shown in Supplementary Figure 12.

Plant traits investigation

Plant traits data were obtained from a large-scale field survey when biomass peaked during the growing season from 2019 to 2021 on the Qinghai-Tibetan Plateau. In brief, 1564 sites were surveyed by using a rasterized sampling method. These sites were distributed with a 0.5° grid size in latitudinal and longitudinal directions. In each site, plant samples for each species of were collected from three plant communities, in addition, plant samples of all visible species within 1 km area around the sample site were also collected. 20 mature leaves of each plant species were collected for the measurements of plant traits. Details of the field survey and method are described in Li et al. (2022) [82], Wang et al. (2021) [83] and Zhang et al. (2020) [84]. We used available plant traits that related to ecosystem C uptake or sequestration from this field trait survey. For example, chlorophyll content captures light energy and converts CO_2 and light into organic C compounds [85,86]. Leaf C content represents plant's investment in photosynthetic tissues and refers to the amount of C stored in leaves which can contribute to soil C sequestration [87,88]. The leaf area index provides an estimate of the surface area available for photosynthesis [17,89]. Stomata regulate gas exchanges on the

surface of leaves [90,91]. Larger stomatal size would promote CO_2 uptake while smaller stomata exhibit faster opening and closing rates, enabling shorter plants to respond rapidly to light fluctuations beneath the canopy and efficiently maximize their photosynthesis [90-95]. All these traits are associated with ecosystem C dynamics.

Stomatal size measurement

In brief, 6-8 leaves of each species were cut into 0.5 cm pieces along the main vein and fixed in formalin – acetic acid – alcohol (FAA) solution. Scanning electron microscope (S-3400N, Hitachi, Japan) was used to observe stomatal morphological parameters. Three replicates of pieces for each species were photographed. Stomatal guard cell length (L, μm) and guard cell width (W, μm) were measured by using MIPS software (Optical Instrument Co., Ltd., Chongqing, China). The measurement details are described in Liu et al. (2018) [96]. Stomatal size (S, μm^2) was calculated as:

$$S = \frac{\pi}{4} \times L \times W \quad (2)$$

Chlorophyll content measurement

For the measurement of chlorophyll, 0.1 g fresh leaves were extracted three times by 95% ethanol with four replicates for each plant species. The filtered extraction solutions were adjusted to 50 ml. Then, the chlorophyll content (chlorophyll *a* and chlorophyll *b*) of the solution was measured by using a spectrophotometer (Pharma Spec, UV-1700, Shimadzu, Japan). Based on the Lambert-Beer law, the relationships of the optical density with chlorophyll concentration are calculated as:

$$D_{649} = 24.54\ C_a + 44.24\ C_b \quad (3)$$

$$D_{665} = 83.31\ C_a + 18.60\ C_b \quad (4)$$

$$C = C_a + C_b \quad (5)$$

$$Chl = \frac{C \times 50}{100 \times (1 - W\%)} \quad (6)$$

where D_{649} and D_{665} are the optical densities of the chlorophyll solution at wavelengths of 649 and 665 nm; C_a, C_b and C represent the concentrations of chlorophyll a, chlorophyll b and total chlorophyll (g L^{-1}) in the solution; the coefficients 24.54 and 44.24 are the specific absorption of chlorophyll a and chlorophyll b at the wavelength of 649 nm, 83.31 and 18.60 are at 665 nm. Chl is the total chlorophyll content per gram of dry weight (mg g^{-1}) and $W\%$ is the percentage of species leaf water content. Details on the measurement are described in Li et al. (2018) [97] and Zhang et al. (2020) [98].

Leaf C content measurement

The dried leaf samples were finely ground by an agate mortar grinder (RM200, Retsch, Haan, Germany) with a ball mill (MM400 Ball Mill, Retsch). Then, we used an elemental analyzer (Vario Max CN Element Analyzer, Elementar, Hanau, Germany) to measure leaf C content (%) [84]. Plant functional traits at the community level were calculated as the same as community-weighted height. In the warming experiment, we used the trait values collected from the field survey under natural condition weighted by community compositional data in the warming communities, we estimated changes in plant functional traits (stomatal size, chlorophyll content and leaf C content) at the community level in the warming plots, assuming that changes in community weighted traits were mainly caused by species turnover rather than intraspecific trait variation among warming treatments [21,24]. The relationships of community weighted height with

other plant functional traits at the community level are shown in Figures 2 and 4.

Obtaining leaf area index and net ecosystem productivity from remote-sensed data

The MODIS (Moderate Resolution Imaging Spectroradiometer) provides satellite-based observations of carbon fluxes and surface measurements from 1982 to present [99,100]. We extracted leaf area index (LAI) for each site of 1 km spatial resolution, and 8-day temporal resolution from LPDAAC. The daily net ecosystem productivity (NEP) data was extracted from SMAP (soil moisture active passive) level-4 NEE product with a 9-km resolution [101]. To be consistent with the timing of our transect study, annual average LAI and NEP in 2019 for each site were calculated.

Factors controlling the variation in NEP

We used structural equation model (SEM) to estimate the pathways through which warming influenced the NEP in both the warming experiment and the transect study (Supplementary Fig. 16). In the warming experiment, the biotic and abiotic factors included community weighted height, community weighted chlorophyll content, community weighted stomatal size, community weighted leaf C content, mean soil temperature during the growing season and mean annual precipitation. In the transect study, the factors included community weighted height, community weighted height chlorophyll content, community weighted height stomatal size, community weighted height leaf C content, leaf area index, mean annual temperature, mean annual precipitation, soil available P and soil total N. All potential causal variables and relationships were considered in a priori model, and we simplified the model by removing redundant variables and non-significant pathways. Model performance was

assessed through Chi-square (χ^2) test, root mean square error of approximation (RMSEA) and Akaike information criterion (AIC). The final optimal mode was selected based on the lowest AIC value. Moreover, we used ridge regression to control for collinearity among covariates and to evaluate the relative importance of each variable on NEP in both the warming experiment and the transect study (Supplementary Fig. 17). The ridge regression was performed using the “ridge (v3.3)” package in R statistical software v 3.4.3 (The R Foundation for Statistical Computing, Vienna, Austria), and the SEM models were conducted using AMOS 21.0 (Amos Development Corporation, Chicago, IL, USA).

Supplementary Tables and Figures

Supplementary Table 1 Repeated measures ANOVA results (two-sided statistical test, F and *P* values) on the effects of warming treatment, year, plant functional type, and their interactions on mean plant height (n = 5).

	Mean plant height	
	F	*P*
Functional type	116.6485	2.2×10^{-16}
Warming	5.9193	0.0032
Year	1.1265	0.3397
Functional type × Warming	1.3477	0.2383
Functional type × Year	6.1356	1.607×10^{-7}
Warming × Year	3.1636	0.0056
Functional type × Warming × Year	1.1301	0.3265

Supplementary Table 2 Information of investigation sites. MAT: mean annual temperature; MAP: mean annual precipitation.

Site	Longitude	Latitude	Altitude (m)	MAT (°C)	MAP (mm)
1	91°30’21”	31°34’20”	4564	-3.47	457.00
2	89°40’47”	31°33’59”	4611	-3.22	459.91
3	80°38’34”	32°23’13”	4772	-2.48	88.16
4	85°27’06”	32°01’15”	4820	-1.64	114.93
5	81°21’44”	32°10’41”	4680	-1.59	100.99
6	91°52’42”	31°34’31”	4631	-1.54	461.47
7	91°59’17”	31°22’06”	4536	-0.89	454.52
8	81°15’06”	32°16’24”	4578	-0.81	95.33
9	82°25’41”	32°28’60”	4465	-0.81	79.74
10	82°03’04”	32°05’05”	4627	-0.80	100.25
11	80°22’57”	32°22’28”	4542	-0.76	92.06
12	90°07’13”	31°26’23”	4772	-0.73	454.26
13	81°49’35”	32°04’17”	4595	-0.67	106.60
14	80°47’51”	32°23’12”	4461	-0.50	88.22
15	89°49’31”	31°29’01”	4633	-0.34	456.49
16	83°23’51”	32°24’24”	4613	-0.34	71.87
17	90°58’59”	31°24’06”	4578	-0.33	452.55
18	91°02’43”	31°28’05”	4588	-0.20	456.06
19	91°07’25”	31°29’42”	4683	0.03	456.27
20	92°55’54”	31°50’52”	4312	0.03	452.72
21	90°47’19”	31°23’29”	4545	0.03	452.72
22	90°40’24”	31°22’49”	4601	0.08	453.12
23	89°57’45”	31°28’51”	4740	0.09	454.30
24	90°14’31”	31°23’10”	4629	0.24	457.21
25	90°22’14”	31°23’04”	4631	0.34	457.86
26	85°05’53”	31°57’38”	4606	0.35	111.53
27	83°09’25”	32°25’53”	4456	0.40	76.31
28	88°03’44”	31°49’45”	4750	0.51	332.06
29	82°20’47”	32°15’29”	4465	0.52	84.87
30	84°16’27”	32°15’43”	4524	0.55	83.54
31	82°56’25”	32°23’06”	4459	0.59	73.89
32	82°48’52”	32°24’43”	4464	0.79	74.46
33	83°36’32”	32°21’12”	4463	0.84	78.23
34	84°28’28”	32°12’37”	4483	0.90	91.06
35	90°31’18”	31°22’21”	4567	0.97	455.71
36	87°10’01”	31°50’03”	4663	1.02	236.21
37	84°40’44”	32°09’40”	4450	1.11	97.71
38	89°34’59”	31°37’20”	4563	1.12	452.20

39	84°48’02”	32°05’10”	4455	1.19	98.18
40	88°20’47”	31°50’22”	4591	1.20	365.50
41	89°14’27”	31°31’41”	4590	1.34	441.44
42	87°51’00”	31°52’07”	4553	1.48	312.06
43	89°04’47”	31°29’36”	4565	1.59	434.57
44	87°38’42”	31°49’55”	4510	1.82	292.21
45	87°28’41”	31°47’33”	4545	1.82	277.59

Supplementary Table 3 Effects of community weighted height, mean soil temperature during the growing season and mean annual precipitation on net ecosystem productivity, and effects of these environmental factors on community weighted height in the controlled warming experiment (n = 60). The effects were estimated by using the linear mixed-effects model with two-sided test.

	Net ecosystem productivity			Community weighted height		
Predictors	Estimates	Confidence interval (95%)	P	Estimates	Confidence interval (95%)	P
Fixed effects						
Intercept	4.120	(0.957, 7.282)	0.0117	27.078	(7.711, 46.446)	0.007
Community weighted height	0.054	(0.013, 0.094)	0.0108			
Mean growing season soil temperature	0.255	(0.110, 0.399)	0.0008	1.975	(1.192, 2.758)	5.3927×10^{-6}
Mean annual precipitation	-0.002	(-0.007, 0.002)	0.3537	-0.0317	(-0.060, -0.003)	0.0294
Random effects						
SD (Block)	3.859×10^{-5}			7.322×10^{-4}		
Model fit						
Marginal R^2	0.505			0.343		
Conditional R^2	0.505			0.343		
AIC	162.065			369.12		
BIC	174.217			379.336		

Supplementary Table 4 Effects of community weighted height, mean annual temperature, mean annual precipitation, soil available P and soil total N on net ecosystem productivity, and effects of these environmental factors on community weighted height in the transect study (n = 45). The effects were estimated by using the linear mixed-effects model with two-sided test.

	Net ecosystem productivity			Community weighted height		
Predictors	Estimates	Confidence interval (95%)	P	Estimates	Confidence interval (95%)	P
Fixed effects						
Intercept	-0.024	(-0.081, 0.032)	0.3939	8.728	(6.182, 11.274)	2.3628×10^{-8}
Community weighted height	0.006	(0.001, 0.011)	0.0177			
Mean annual temperature	0.002	(-0.006, 0.011)	0.5537	0.739	(0.240, 1.238)	0.0047
Mean annual precipitation	-1.407×10^{-5}	(-7.639×10^{-5}, 4.825×10^{-5})	0.6504	-0.008	(-0.011, -0.005)	9.1777×10^{-6}
Soil available P	0.003	(-0.005, 0.012)	0.4348	-0.236	(-0.817, 0.346)	0.4175
Soil total N	0.003	(-0.014, 0.020)	0.6964	-0.426	(-1.539, 0.688)	0.4442
Random effects						
SD (Site)	0.025			1.678		
Model fit						
Marginal R^2	0.29			0.461		
Conditional R^2	0.912			0.934		
AIC	-122.791			202.252		
BIC	-109.483			214.074		

Supplementary Figure 1 Relationships between net ecosystem productivity (NEP) and (a) height variability and (b) changes in plant community composition in the controlled warming experiment. Linear regression with two-sided test was used for the statistical analysis. The coefficient of determination (R^2) and the exact P values for all the regressions were indicated. The error bands are 95% confidence intervals (± 1.96 s.e.m.) around the fitted regression lines, sample size n = 60.

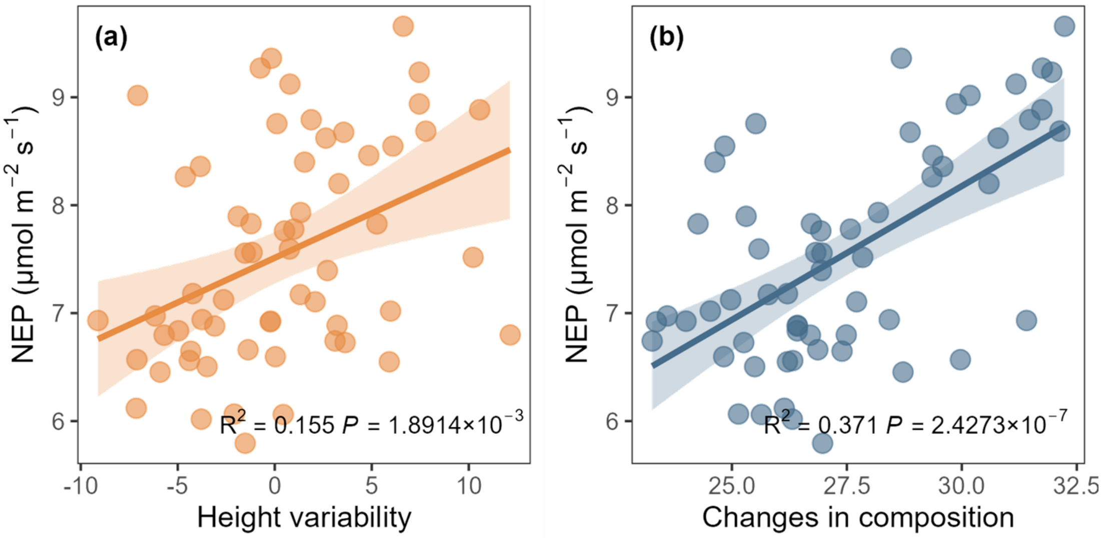

Supplementary Figure 2 Relationships between plant species height and (a) chlorophyll content and (b) stomatal size in the controlled warming experiment. Linear regression with two-sided test was used for the statistical analysis. The coefficient of determination (R^2) and the exact P values for all the regressions were indicated. The error bands are 95% confidence intervals (± 1.96 s.e.m.) around the fitted regression lines, sample size n = 37.

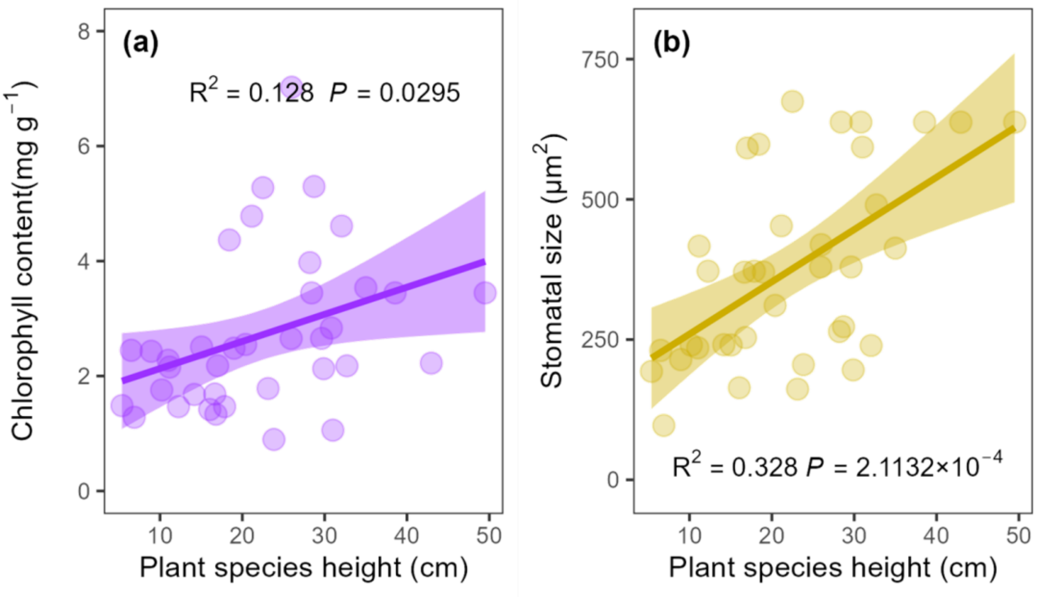

Supplementary Figure 3 Relationships between net ecosystem productivity (NEP) and plant community traits for (a) chlorophyll content, (b) stomatal size, and (c) leaf C content in the controlled warming experiment. Linear regression with two-sided test was used for the statistical analysis. The coefficient of determination (R^2) and the exact *P* values for all the regressions were indicated. The error bands are 95% confidence intervals (± 1.96 s.e.m.) around the fitted regression lines, sample size n = 60.

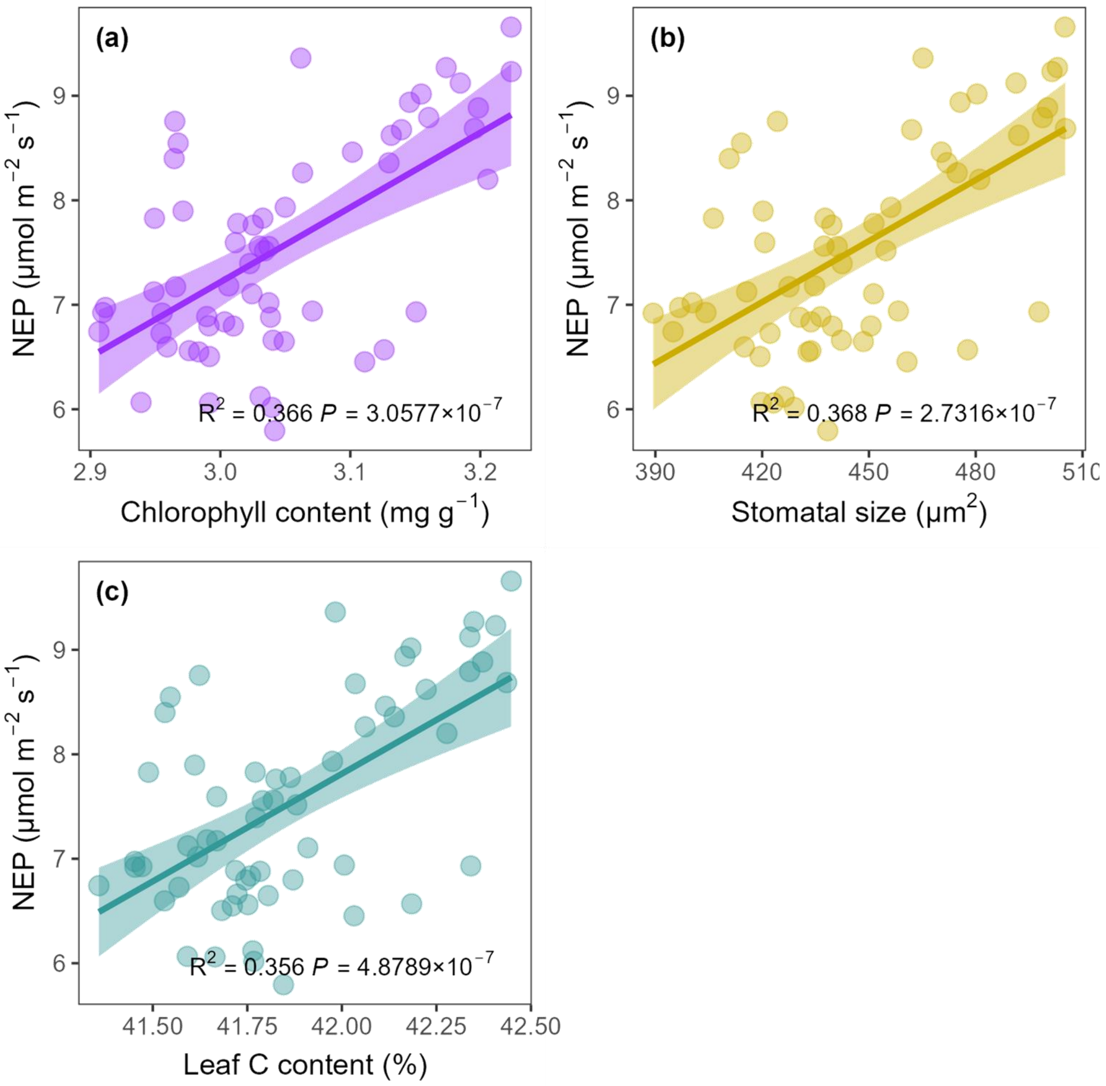

Supplementary Figure 4 Temperature sensitivities of (a) NEP, (b) GEP, and (c) ER changing with community weighted height. The temperature sensitivities are the slopes of rolling regressions between mean soil temperature during the growing season and ecosystem C fluxes along the community weighted height gradient with different (10, 20, 30, 40, 50) rolling width. The error bands are 95% confidence intervals (± 1.96 s.e.m.) around the fitted regression lines, sample size n = 60.

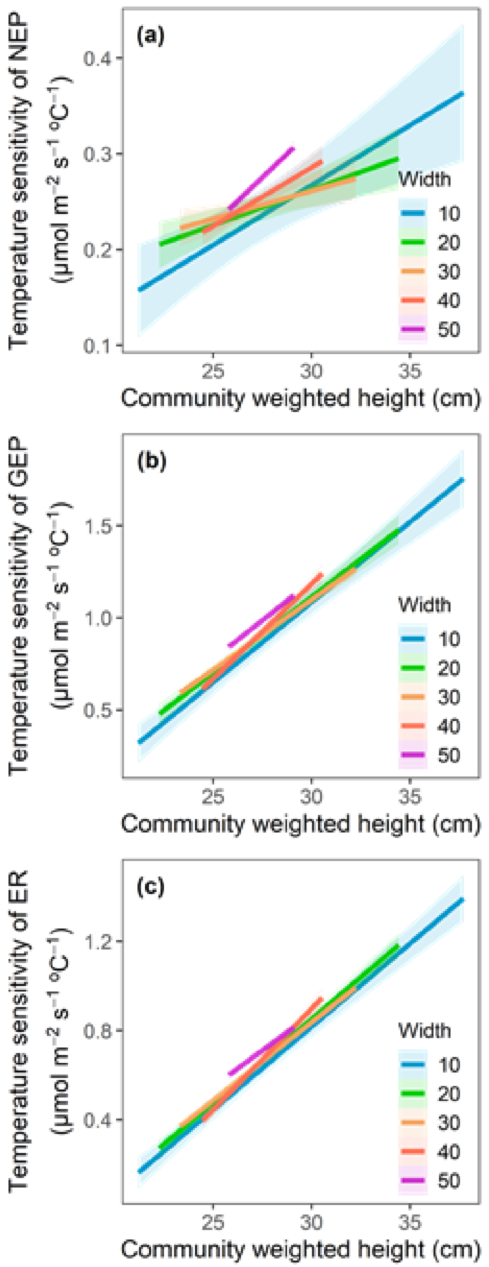

Supplementary Figure 5 Relationships of net ecosystem productivity (NEP) with plant community traits for (a) chlorophyll content, (b) stomatal size, (c) leaf C content, and (d) leaf area index in the transect study. Linear regression with two-sided test was used for the statistical analysis. The coefficient of determination (R^2) and the exact *P* values for all the regressions were indicated. The error bands are 95% confidence intervals (± 1.96 s.e.m.) around the fitted regression lines, sample size n = 45.

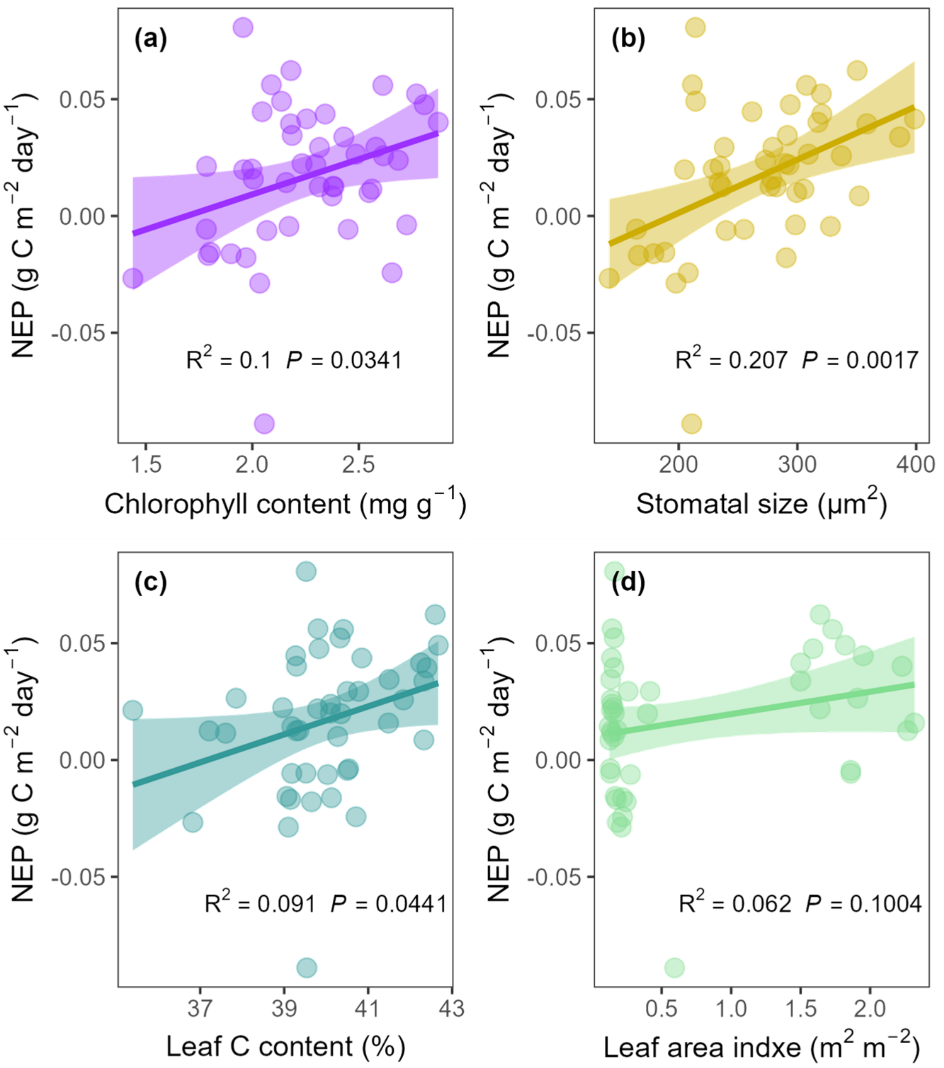

Supplementary Figure 6 Relationships of soil total C content with (a) community weighted height and (b) net ecosystem productivity (NEP) in the transect study. Linear regression with two-sided test was used for the statistical analysis. The coefficient of determination (R^2) and the exact P values for all the regressions were indicated. The error bands are 95% confidence intervals (± 1.96 s.e.m.) around the fitted regression lines, sample size n = 45.

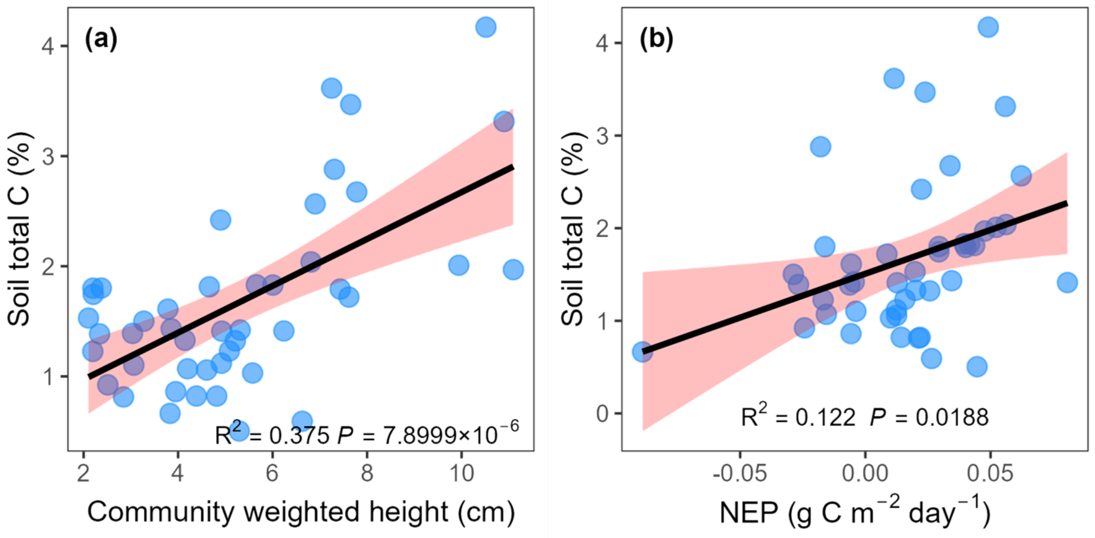

Supplementary Figure 7 Relationships between net ecosystem productivity (NEP) and the proportional above-ground net primary production (ANPP) of grass (a), forbs (b), legumes (c) and sedges (d) in community and the community composition index (CCI) (e) in the controlled warming experiment. Linear regression with two-sided test was used for the statistical analysis. The coefficient of determination (R^2) and the exact *P* values for all the regressions were indicated. The error bands are 95% confidence intervals (± 1.96 s.e.m.) around the fitted regression lines, sample size n = 60.

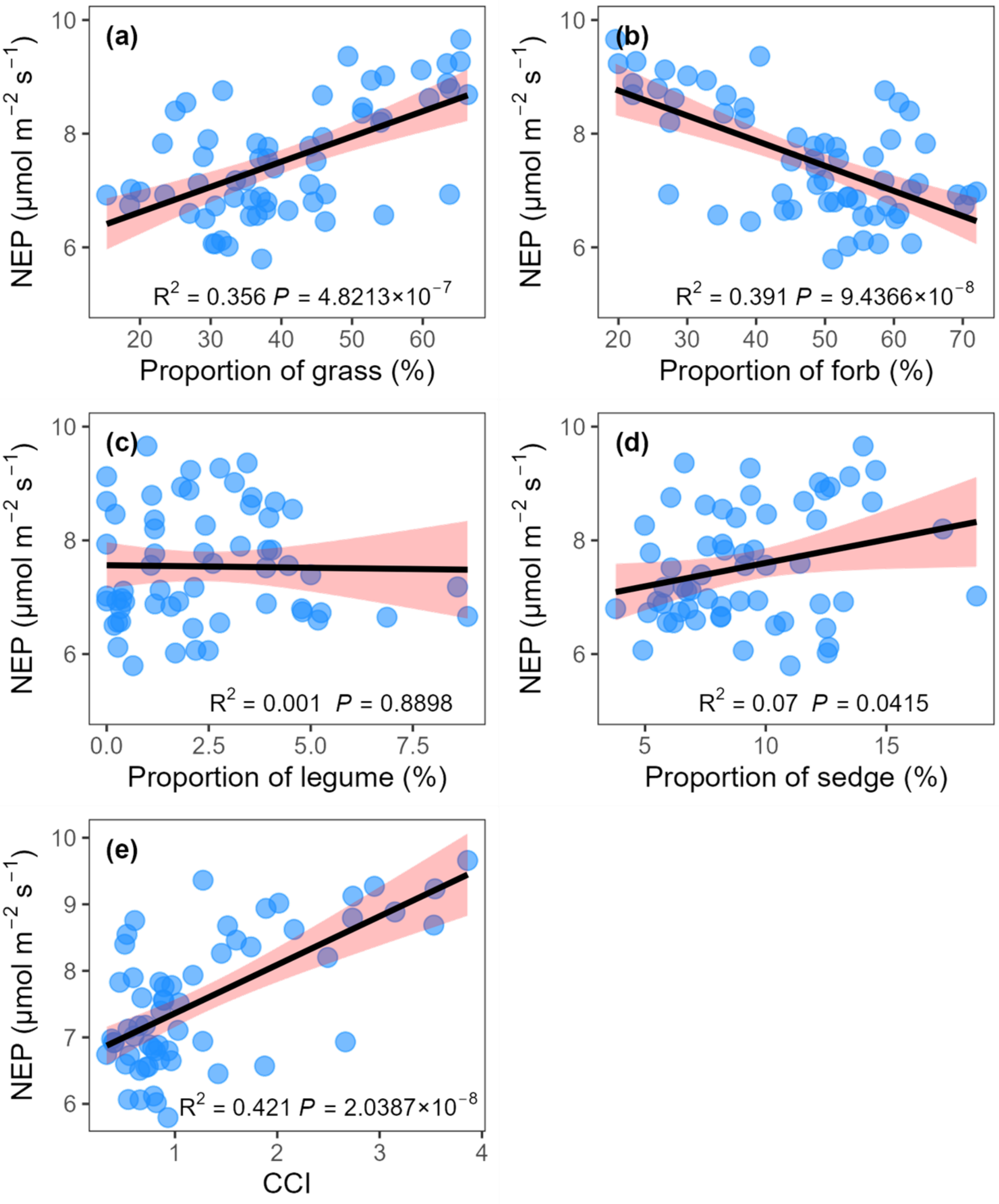

Supplementary Figure 8 Annual dynamics and means of soil total C content from 2015 to 2017 under control (C), low-level warming (W1) and high-level warming (W2) in the controlled warming experiment. Repeated-measures ANOVA with two-sided test was used to determine the significant effects. Significance of the effects were indicated by exact *P* values. Data are presented as mean ± s.e.m., sample size n = 5.

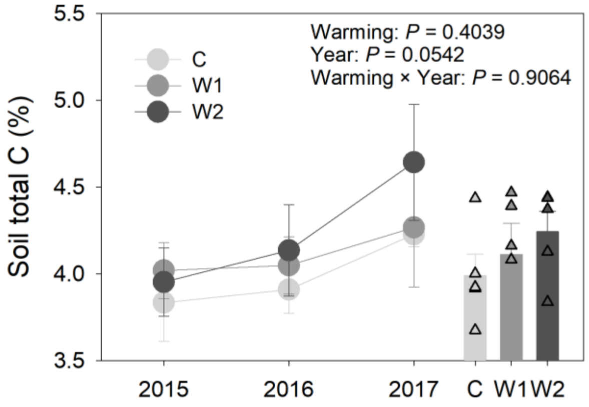

Supplementary Figure 9 Partial relationships of net ecosystem productivity (NEP) with community weighted height (CWH) for controlling the effects of above-ground net primary production (ANPP) (a) in the controlled warming experiment, sample size n = 60, and (b) in the transect study, sample size n = 45. Linear mixed-effect model was used to estimate the partial relationships. The partial regression with two-sided test was used. The coefficient of determination (R^2) and the exact *P* values were indicated. The error bands are 95% confidence intervals (± 1.96 s.e.m.) around the fitted regression lines.

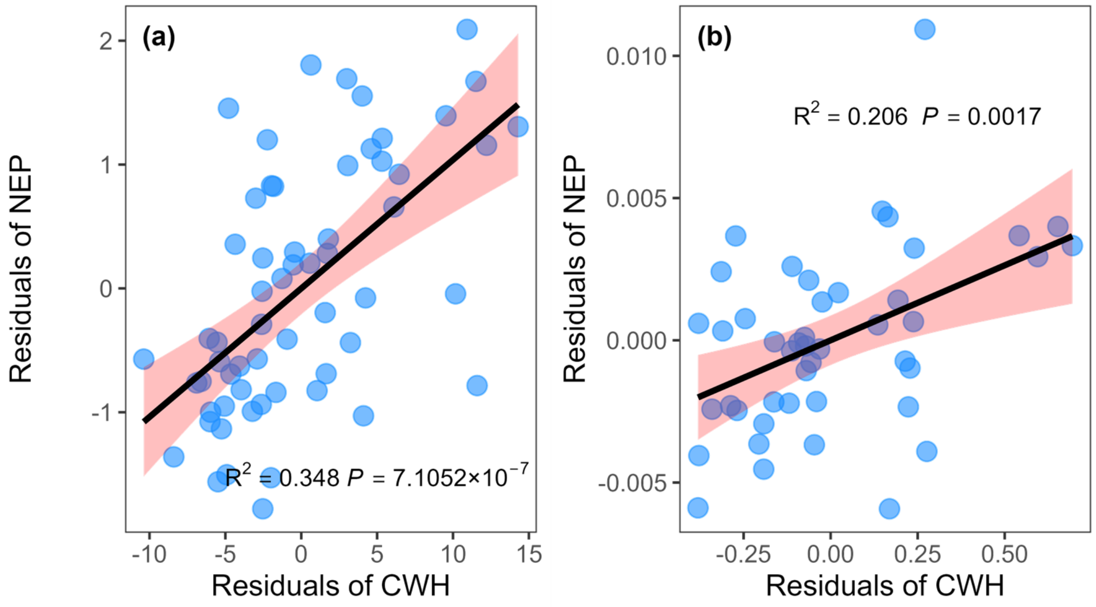

Supplementary Figure 10 Partial relationships of soil total C content with community weighted height (CWH) for controlling the effects of above-ground net primary production in the transect study. Linear mixed-effect model was used to estimate the partial relationship. The partial regression with two-sided test was used. The coefficient of determination (R^2) and the exact *P* values were indicated. The error bands are 95% confidence intervals (± 1.96 s.e.m.) around the fitted regression lines, sample size n = 45.

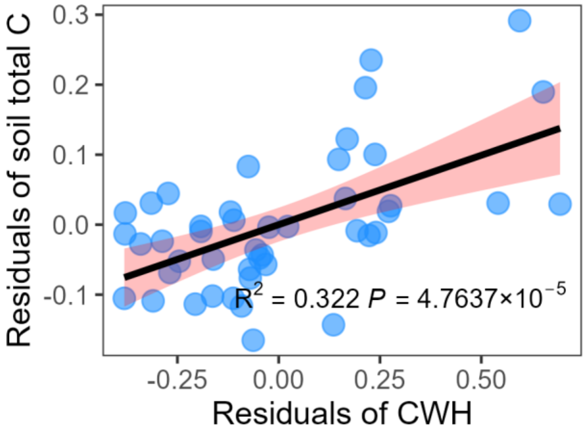

Supplementary Figure 11 Relationships between CWH and net ecosystem productivity (NEP) per unit ANPP (a) in the controlled warming experiment, sample size n = 60, and (b) in the transect study, sample size n = 45. Linear regression with two-sided test was used for the statistical analysis. The coefficient of determination (R^2) and the exact *P* values were indicated. The error bands are 95% confidence intervals (± 1.96 s.e.m.) around the fitted regression lines.

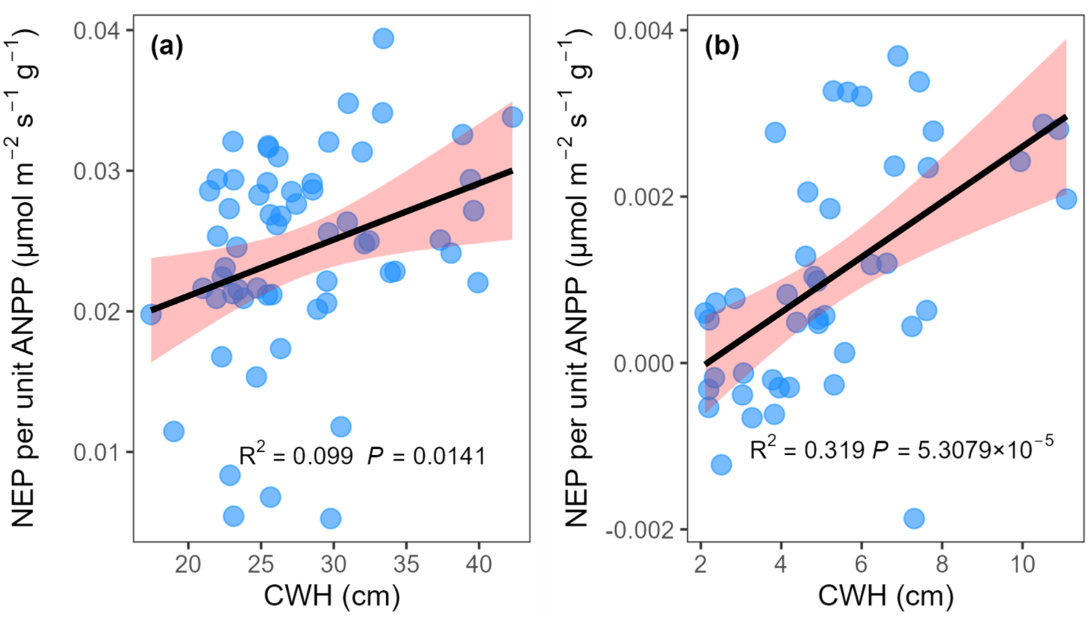

Supplementary Figure 12 Partial relationships of community composition index (CCI) with mean soil temperature during the growing season (ST) for controlling the effects of annual precipitation and block (a) and with annual precipitation for controlling the effects of ST and block (b) in the warming experiment. Linear mixed-effect model was used to estimate the partial relationship. The partial regression with two-sided test was used. The coefficient of determination (R^2) and the exact *P* values were indicated. The error bands are 95% confidence intervals (± 1.96 s.e.m.) around the fitted regression lines, sample size n = 60.

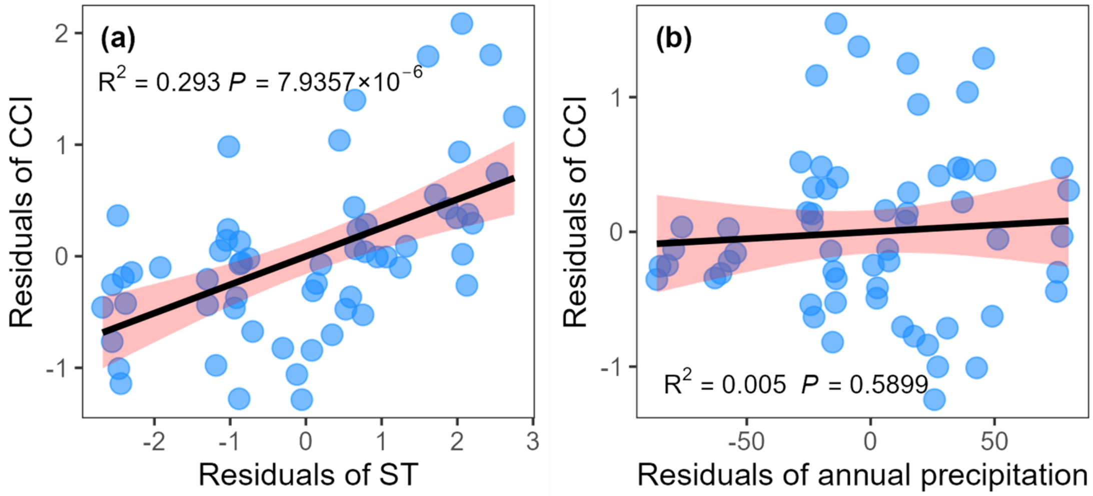

Supplementary Figure 13 Light intensity of different height from ground surface (0 cm) to canopy (50 cm) of plant community in the control plots. Data are presented as mean ± s.e.m., sample size n = 4 for 0 cm to 40 cm and n = 3 for 50 cm. One-way ANOVA followed by two-sided LSD test was used for multiple comparison. Different letters close to the bars indicate significant differences among height levels at α = 0.05.

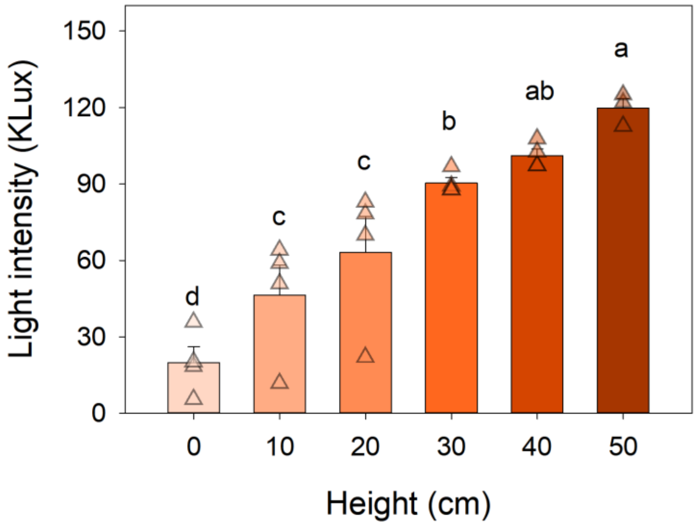

Supplementary Figure 14 Partial relationships of net ecosystem productivity (NEP) (a), gross ecosystem productivity (GEP) (b) and ecosystem respiration (ER) (c) with mean soil temperature during the growing season (ST, red lines) for controlling the effects of community composition index (CCI) and block, and with CCI (blue lines) for controlling the effects of ST and block, in the controlled warming experiment. Linear mixed-effect model was used to estimate the partial relationship. The partial regression with two-sided test was used. The coefficient of determination (R^2) and the exact *P* values were indicated. The error bands are 95% confidence intervals (± 1.96 s.e.m.) around the fitted regression lines, sample size n = 60. Different letters close to the slopes indicate significant differences among slopes (analysis of covariance, ANCOVA with two-sided test).

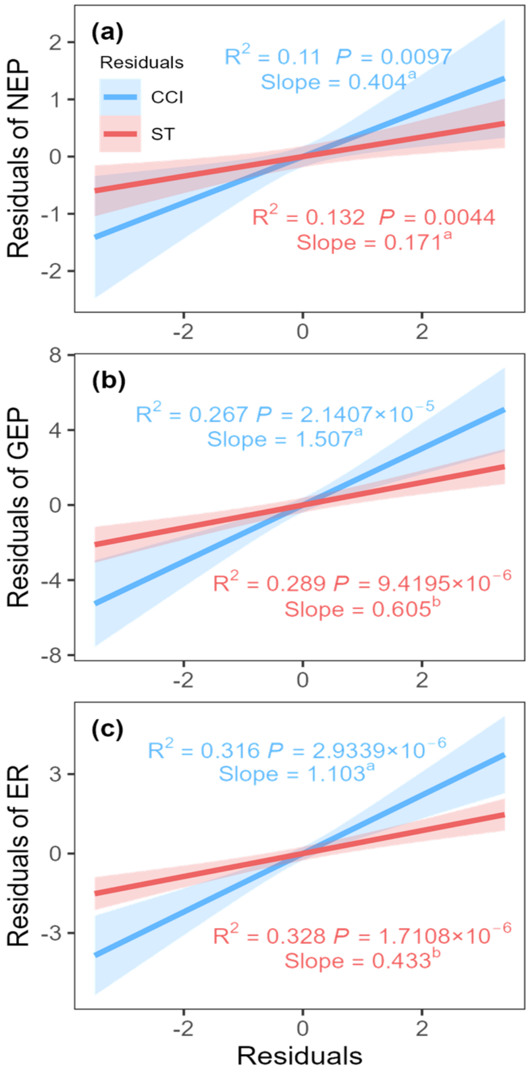

Supplementary Figure 15 (a) Partial relationship of community weighted height (CWH) with mean soil temperature during the growing season (ST) for controlling the effects of mean annual precipitation and block in the controlled warming experiment. (b) Partial relationship of net ecosystem productivity (NEP) with CWH for controlling the effects of ST, mean annual precipitation and block in the controlled warming experiment. Linear mixed-effect model was used to estimate the partial relationship. The partial regression with two-sided test was used. The coefficient of determination (R^2) and the exact P values were indicated. The error bands are 95% confidence intervals (± 1.96 s.e.m.) around the fitted regression lines, sample size n = 60.

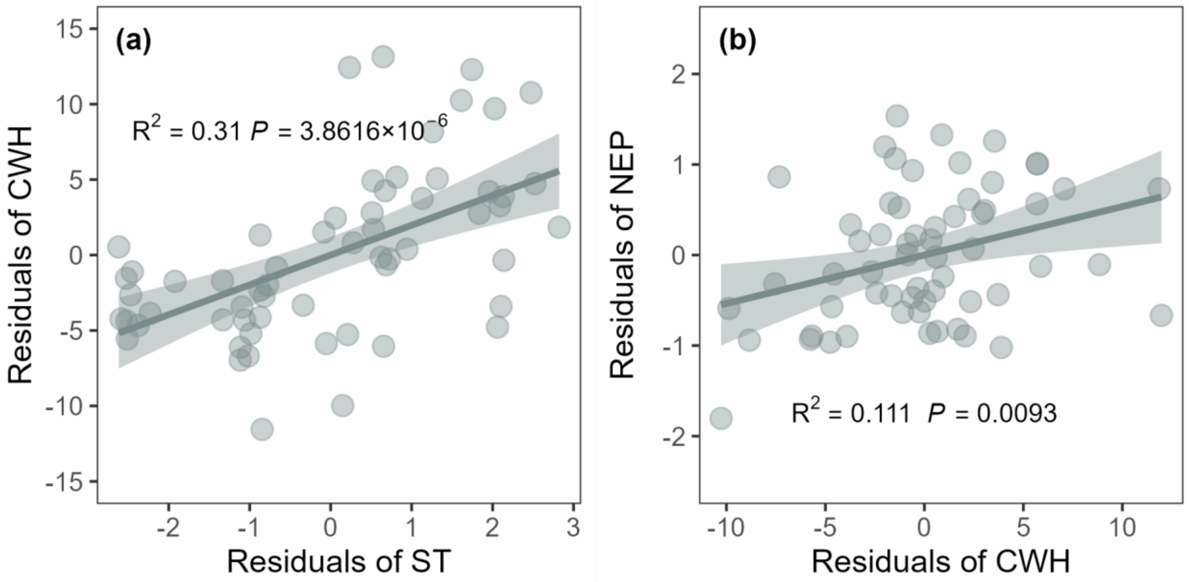

Supplementary Figure 16 The results of the structural equation model showing the direct and indirect effects of warming on net ecosystem productivity in the warming experiment (n = 60) (a) and (b) in the transect study (n = 45). Brown and black solid arrows represent significant positive and negative pathways, respectively. The values adjacent to the arrows are standardized path coefficients, which reflect the effect size of the relationship. The width of the arrow width is proportional to the strength of the relationship. R^2 values associated with variables indicate the proportion of variation explained by relationships with other variables. Level of significance is indicated by: *** $P < 0.001$; **$0.001 \leq P < 0.01$; *$0.01 \leq P < 0.05$. H: community-weighted height; SS: community-weighted stomatal size; CHL: community-weighted chlorophyll content; LC: community-weighted leaf C content; LAI: leaf area index; Tem: mean growing season soil temperature in panel (a) and mean annual temperature in panel (b); Pre: mean annual precipitation; AP: soil available phosphorus; TN: soil total nitrogen. Goodness-of-fit statistics for (a): $\chi^2 = 10.138$, $P = 0.119$, df = 6, root mean square error of approximation (RMSEA) = 0.108, AIC = 54.138; for (b): $\chi^2 = 11.565$, $P = 0.951$, df = 21, RMSEA < 0.001, AIC = 59.565.

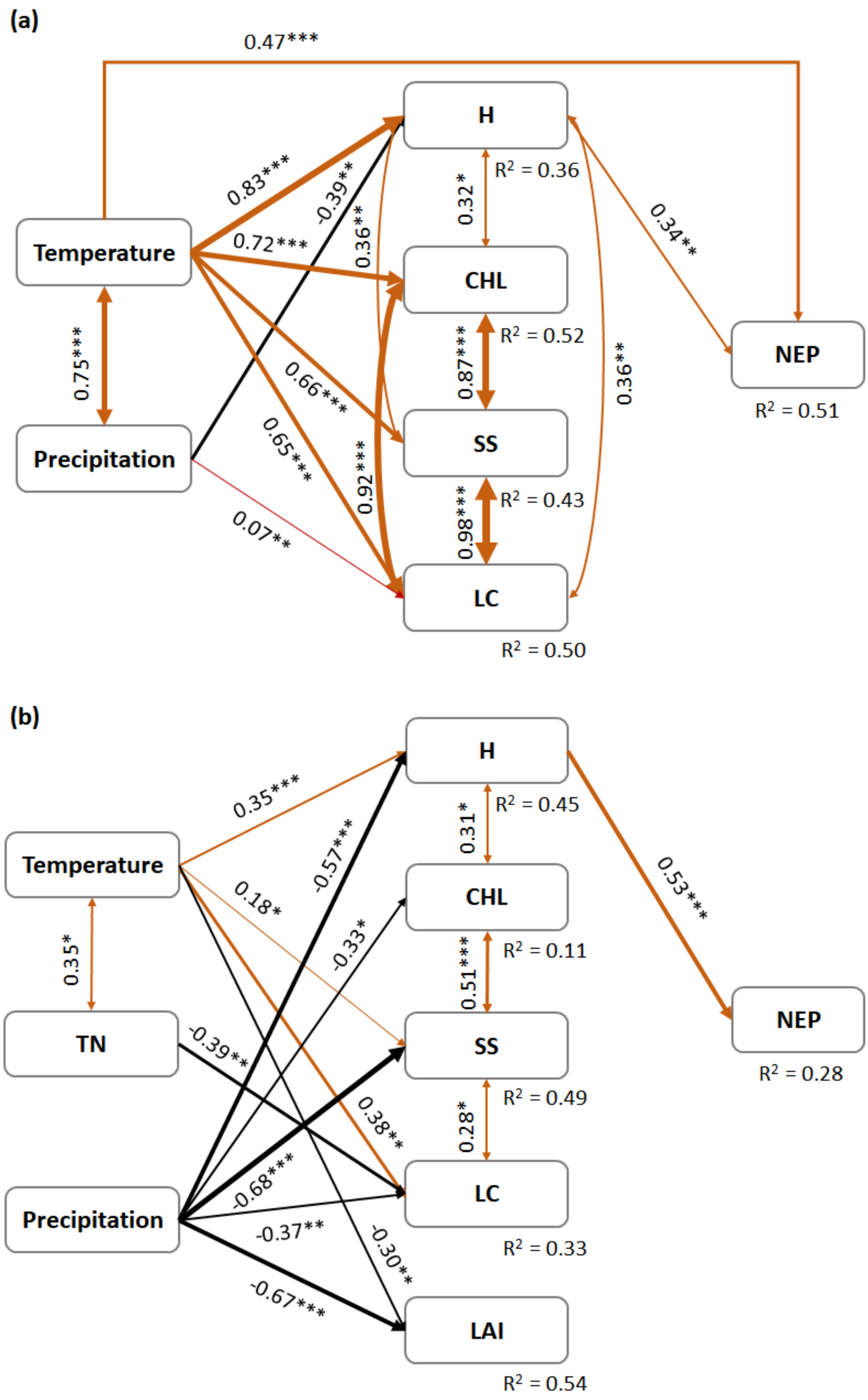

(a)
0.47***
H
CHL
SS
LC
NEP
Temperature
Precipitation
0.83***
-0.39**
0.72***
0.36**
0.32*
R² = 0.36
0.34**
0.75***
0.87***
R² = 0.52
0.36**
R² = 0.51
0.66***
0.65***
0.92***
R² = 0.43
0.98***
0.07**
R² = 0.50
(b)
H
CHL
SS
LC
LAI
NEP
Temperature
TN
Precipitation
0.35***
-0.57***
R² = 0.45
0.31*
0.53***
0.18*
-0.33*
R² = 0.11
0.51***
0.35*
R² = 0.28
-0.39**
0.38**
R² = 0.49
0.28*
-0.68***
-0.37**
R² = 0.33
-0.30**
-0.67***
R² = 0.54

Supplementary Figure 17 The response of net ecosystem productivity (NEP) to influencing variables in the warming experiment (n = 60) (a) and the transect study (n = 45) (b). H: community-weighted height; SS: community-weighted stomatal size; CHL: community-weighted chlorophyll content; LC: community-weighted leaf C content; LAI: leaf area index; Tem: mean growing season soil temperature in panel (a) and mean annual temperature in panel (b); Pre: mean annual precipitation; AP: soil available phosphorus; TN: soil total nitrogen. Circles indicate the means of scaled estimates of the ridge regression between NEP and the variables, which reflect the importance of variables. Error bars represent 95% confidence intervals.

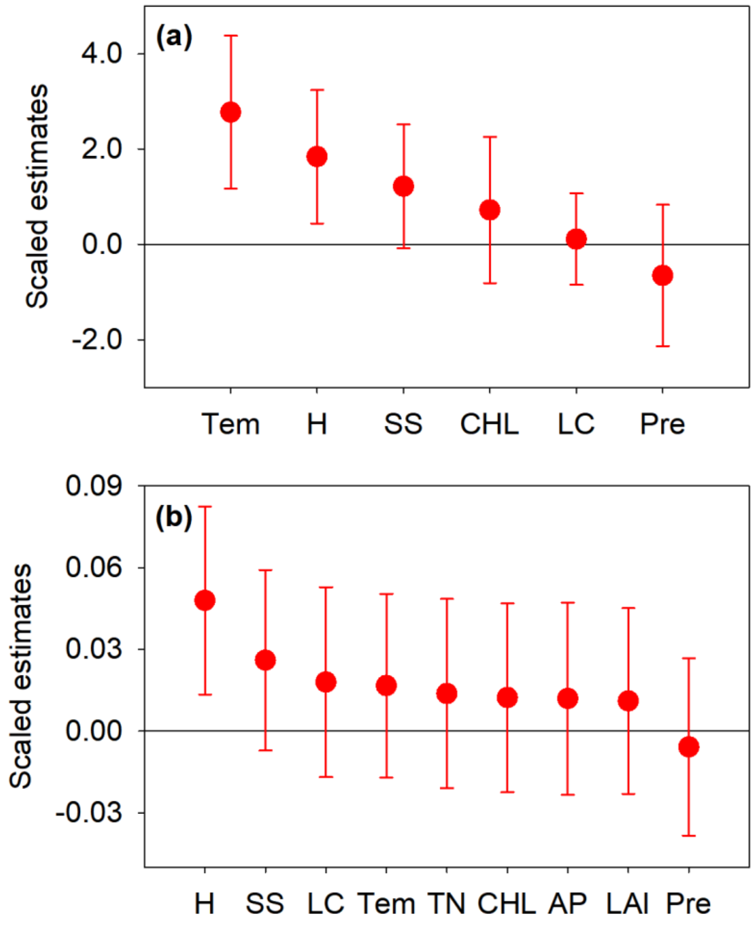

81 Leps, J., de Bello, F., Smilauer, P. & Dolezal, J. Community trait response to environment: disentangling species turnover vs intraspecific trait variability effects. *Ecography* **34**, 856-863, doi:10.1111/j.1600-0587.2010.06904.x (2011).
82 Li, X. *et al.* Allometry and Distribution of Nitrogen in Natural Plant Communities of the Tibetan Plateau. *Front Plant Sci* **13** (2022).
83 Wang, R. M., He, N. P., Li, S. G., Xu, L. & Li, M. X. Variation and adaptation of leaf water content among species, communities, and biomes. *Environ Res Lett* **16** (2021).
84 Zhang, J. H. *et al.* Variation and evolution of C:N ratio among different organs enable plants to adapt to N-limited environments. *Global Change Biol* **26**, 2534-2543, doi:10.1111/gcb.14973 (2020).
85 Croft, H. *et al.* Leaf chlorophyll content as a proxy for leaf photosynthetic capacity. *Global Change Biol* **23**, 3513-3524, doi:10.1111/gcb.13599 (2017).
86 Luo, X. Z., Croft, H., Chen, J. M., He, L. M. & Keenan, T. F. Improved estimates of global terrestrial photosynthesis using information on leaf chlorophyll content. *Global Change Biol* **25**, 2499-2514, doi:10.1111/gcb.14624 (2019).
87 Webster, E. A., Chudek, J. A. & Hopkins, D. W. Carbon transformations during decomposition of different components of plant leaves in soil. *Soil Biol Biochem* **32**, 301-314, doi:Doi 10.1016/S0038-0717(99)00153-4 (2000).
88 Garnier, E. *et al.* Plant functional markers capture ecosystem properties during secondary succession. *Ecology* **85**, 2630-2637, doi:Doi 10.1890/03-0799 (2004).
89 Falster, D. S., Duursma, R. A. & FitzJohn, R. G. How functional traits influence plant growth and shade tolerance across the life cycle. *P Natl Acad Sci USA* **115**, E6789-E6798, doi:10.1073/pnas.1714044115 (2018).
90 Henry, C. *et al.* A stomatal safety-efficiency trade-off constrains responses to leaf dehydration. *Nat Commun* **10** (2019).
91 Franks, P. J., Drake, P. L. & Beerling, D. J. Plasticity in maximum stomatal conductance constrained by negative correlation between stomatal size and density: an analysis using Eucalyptus globulus. *Plant Cell Environ* **32**, 1737-1748, doi:10.1111/j.1365-3040.2009.002031.x (2009).
92 Drake, P. L., Froend, R. H. & Franks, P. J. Smaller, faster stomata: scaling of stomatal size, rate of response, and stomatal conductance. *J Exp Bot* **64**, 495-505, doi:10.1093/jxb/ers347 (2013).
93 Pearcy, R. W. Sunflecks and photosynthesis in plant canopies. *Annu Rev Plant Phys* **41**, 421-453, doi:10.1146/annurev.pp.41.060190.002225 (1990).
94 McAusland, L. *et al.* Effects of kinetics of light-induced stomatal responses on photosynthesis and water-use efficiency. *New Phytol* **211**, 1209-1220, doi:10.1111/nph.14000 (2016).
95 Xiong, Z. *et al.* Effect of Stomatal Morphology on Leaf Photosynthetic Induction Under Fluctuating Light in Rice. *Front Plant Sci* **12** (2022).
96 Liu, C. C. *et al.* Variation of stomatal traits from cold temperate to tropical forests and association with water use efficiency. *Funct Ecol* **32**, 20-28, doi:10.1111/1365-2435.12973 (2018).
97 Li, Y. *et al.* Variation in leaf chlorophyll concentration from tropical to cold-temperate forests: Association with gross primary productivity. *Ecol Indic* **85**, 383-389, doi:10.1016/j.ecolind.2017.10.025 (2018).

98 Zhang, Y. *et al.* Spatial Variation of Leaf Chlorophyll in Northern Hemisphere Grasslands. *Front Plant Sci* **11** (2020).
99 Myneni, R., Knyazikhin, Y. & Park, T. J. N. E. L. P. D. MOD15A2H MODIS/Terra leaf area Index/FPAR 8-Day L4 global 500m SIN grid V006. (2015).
100 Running, S. W. & Zhao, M. J. M. U. s. G. Daily GPP and annual NPP (MOD17A2/A3) products NASA Earth Observing System MODIS land algorithm. **2015**, 1-28 (2015).
101 Kimball, J., Jones, L., Kundig, T. & Reichle, R. SMAP L4 Global Daily 9 km EASE-Grid Carbon Net Ecosystem Exchange, Version 4. Boulder, Colorado USA. *NASA National Snow and Ice Data Center Distributed Active Archive Center* (2018).
