# Supplementary material for: Plant height as an indicator for alpine carbon sequestration and ecosystem response to warming
Source: Nat Plants. 2024 May 16;10(6):890–900. doi: 10.1038/s41477-024-01705-z (PMC11208140; doi:10.1038/s41477-024-01705-z)
Supplement: Supplementary file 2 — Reporting Summary [file 41477_2024_1705_MOESM2_ESM.pdf]

nature portfolio

Corresponding author(s): Shuli Niu
Last updated by author(s): Mar 29, 2024

Reporting Summary

Nature Portfolio wishes to improve the reproducibility of the work that we publish. This form provides structure for consistency and transparency in reporting. For further information on Nature Portfolio policies, see our Editorial Policies and the Editorial Policy Checklist.

Statistics

For all statistical analyses, confirm that the following items are present in the figure legend, table legend, main text, or Methods section.

n/a	Confirmed	
☐	☒	The exact sample size (*n*) for each experimental group/condition, given as a discrete number and unit of measurement
☐	☒	A statement on whether measurements were taken from distinct samples or whether the same sample was measured repeatedly
☐	☒	The statistical test(s) used AND whether they are one- or two-sided<br>*Only common tests should be described solely by name; describe more complex techniques in the Methods section.*
☒	☐	A description of all covariates tested
☒	☐	A description of any assumptions or corrections, such as tests of normality and adjustment for multiple comparisons
☐	☒	A full description of the statistical parameters including central tendency (e.g. means) or other basic estimates (e.g. regression coefficient) AND variation (e.g. standard deviation) or associated estimates of uncertainty (e.g. confidence intervals)
☐	☒	For null hypothesis testing, the test statistic (e.g. *F*, *t*, *r*) with confidence intervals, effect sizes, degrees of freedom and *P* value noted<br>*Give P values as exact values whenever suitable.*
☒	☐	For Bayesian analysis, information on the choice of priors and Markov chain Monte Carlo settings
☒	☐	For hierarchical and complex designs, identification of the appropriate level for tests and full reporting of outcomes
☒	☐	Estimates of effect sizes (e.g. Cohen's *d*, Pearson's *r*), indicating how they were calculated

Our web collection on statistics for biologists contains articles on many of the points above.

Software and code

Policy information about availability of computer code

Data collection	Stomatal morphological parameters: MIPS software (Optical Instrument Co., Ltd., Chongqing, China)
Data analysis	We used the open source programming language R version 3.4.3 for all data analysis. Analyses utilized R packages 'visreg (v2.7.0)', ' zoo (v1.8-12)', 'nlme (v3.1-163)' and 'ridge (v3.3)'.

For manuscripts utilizing custom algorithms or software that are central to the research but not yet described in published literature, software must be made available to editors and reviewers. We strongly encourage code deposition in a community repository (e.g. GitHub). See the Nature Portfolio guidelines for submitting code & software for further information.

Data

Policy information about availability of data

All manuscripts must include a data availability statement. This statement should provide the following information, where applicable:

- Accession codes, unique identifiers, or web links for publicly available datasets
- A description of any restrictions on data availability
- For clinical datasets or third party data, please ensure that the statement adheres to our policy

All data reported in this paper have been deposited in Figshare Digital Repository (https://doi.org/10.6084/m9.figshare.23519208)

Research involving human participants, their data, or biological material

Policy information about studies with human participants or human data. See also policy information about sex, gender (identity/presentation), and sexual orientation and race, ethnicity and racism.

Reporting on sex and gender	NA
Reporting on race, ethnicity, or other socially relevant groupings	NA
Population characteristics	NA
Recruitment	NA
Ethics oversight	NA

Note that full information on the approval of the study protocol must also be provided in the manuscript.

Field-specific reporting

Please select the one below that is the best fit for your research. If you are not sure, read the appropriate sections before making your selection.

☐ Life sciences ☐ Behavioural & social sciences ☒ Ecological, evolutionary & environmental sciences

For a reference copy of the document with all sections, see nature.com/documents/nr-reporting-summary-flat.pdf

Ecological, evolutionary & environmental sciences study design

All studies must disclose on these points even when the disclosure is negative.

Study description	Combining a field manipulative warming experiment and a regional transect investigation, as well as a large-scale regional sampling of plant functional traits across the high-altitude Qinghai-Tibet Plateau (QTP), we studied how warming-induced changes in grassland community composition and height alter ecosystem C sequestration in this high-altitude, cold region. A randomized complete block design with three warming treatments (control, low-level warming +1.5°C and high-level warming +2.5°C) and five replications was used in the warming experiment.
Research sample	Net ecosystem productivity, ecosystem respiration, plant community composition and plant height of each species were measured in the warming experiment. In the regional transect investigation, we measured plant community composition, plant height of each species and soil total carbon content. Plant traits including stomatal size, chlorophyll content, and leaf carbon content for each species, were obtained from a large-scale field sampling across the QTP. NEP and LAI were obtained from remote sensing product.
Sampling strategy	In the manipulative warming experiment, all data were measured in each plot within each block. Along the regional transect, 45 study sites were investigated, in each site, 10 quadrats were randomly selected for data collection. In the regional plant traits sampling, 1546 sites were surveyed by using a rasterized sampling method. These sites were distributed with a 0.5° grid size in latitudinal and longitudinal directions. In each site, plant samples for each species were collected from three plant communities, in addition, plant samples of all visible species within 1 km area around the sample site were also collected.
Data collection	Quan Quan, Fangfang Ma and Bing Song led the data collection of the warming experiment. Ecosystem carbon fluxes were measured with an infrared gas analyzer (LI-6400XT, LI-COR Environmental, Lincoln, Nebraska, USA) attached to the transparent canopy chamber. To investigate the plant community composition, above-ground net primary production (ANPP) was measured by clipping all living plants at the ground level in a 0.1 × 1 m quadrat in each plot. All plants were sorted to species and oven-dried at 65°C for 48 h and weighed. Plant height of each species were measured in each plot in a permanent 0.5 × 0.5 m quadrat, which is at least 30 cm away from the plot edges. Ruiyang Zhang, Junxiao Pan and Jingsong Wang led the data collection of the transect investigation. 45 study sites along a 1500 km transect in the QTP grasslands was conducted during the peak growing period. In each site, 10 0.5 × 0.5 m quadrats were randomly selected to measure plant community composition, ANPP and plant species height, the methods were the same as those used in the warming experiment. Soil samples were randomly collected from three of the 10 quadrats by using a 7.5 cm diameter soil auger at the depth of 10 cm. Then, soil samples were air-dried and sieved with 2 mm mesh to remove stones and plant roots. Yiheng Wang collected the NEP and LAI from remote sensing product for the transect study. Nianpeng He, Ruomeng Wang, Congcong Liu and Jiahui Zhang led the plant traits sampling. 20 mature leaves of each plant species were collected for the measurements of plant traits.
Timing and spatial scale	In the warming experiment, ecosystem C fluxes were measured twice per month from June 2014 to September 2017 over the growing season (from May to September). Plant community composition, above-ground net primary production and plant height of were measured in the middle of August every year from 2014 to 2017 when biomass peaked. The regional transect investigation was conducted along a 1500 km transect in the QTP grasslands during peak growing period from July to August in 2019. The field survey of plant traits was conducted during peak growing period from 2019 to 2021 on the QTP. 1564 sites were surveyed distributed with a 0.5° rasterized grid size in latitudinal and longitudinal directions.

Data exclusions	None.
Reproducibility	To ensure study reproducibility, we used open source and freely available programming languages. The datasets are available from the figshare Digital Repository. The locations for the warming experiment site, transect investigation sites and plant traits survey sites were given by GPS coordinates or literature references, so that all sites can be surveyed repeatedly by different data collectors.
Randomization	In the warming experiment, plots were randomly assigned to three warming treatments. Quadrats were randomly selected for plant community composition measurement and soil sampling. In the transect investigation, 10 quadrats were randomly selected to measure plant community composition in each site. Leaves of each plant species were also randomly selected to measure plant traits.
Blinding	No blinding was used.

Did the study involve field work? ☒ Yes ☐ No

Field work, collection and transport

Field conditions	In the warming experiment site, the mean annual precipitation is 753 mm and mean annual temperature is 1.1°C, the altitude is 3500 m. The field transect investigation includes 45 study sites, The lowest mean annual temperature among all sites was -3.5°C and the highest was 1.8°C. The lowest mean annual precipitation was 71.9 mm and the highest was 461.5 mm. The average altitude was 4576.2 m. For the regional plant traits survey, the average altitude is over 4000 m, and the average annual temperature is below 0°C in the QTP region.
Location	The Qinghai-Tibet Plateau, China. The average altitude is over 4000 m.
Access & import/export	All access of study sites and data collection complied with local, national and international laws.
Disturbance	Field work resulted in no disturbance.

Reporting for specific materials, systems and methods

We require information from authors about some types of materials, experimental systems and methods used in many studies. Here, indicate whether each material, system or method listed is relevant to your study. If you are not sure if a list item applies to your research, read the appropriate section before selecting a response.

Materials & experimental systems

n/a	Involved in the study	
☒	☐	Antibodies
☒	☐	Eukaryotic cell lines
☒	☐	Palaeontology and archaeology
☒	☐	Animals and other organisms
☒	☐	Clinical data
☒	☐	Dual use research of concern
☐	☒	Plants

Methods

n/a	Involved in the study	
☒	☐	ChIP-seq
☒	☐	Flow cytometry
☒	☐	MRI-based neuroimaging

Dual use research of concern

Policy information about dual use research of concern

Hazards

Could the accidental, deliberate or reckless misuse of agents or technologies generated in the work, or the application of information presented in the manuscript, pose a threat to:

No	Yes	
☒	☐	Public health
☒	☐	National security
☒	☐	Crops and/or livestock
☒	☐	Ecosystems
☒	☐	Any other significant area

Experiments of concern

Does the work involve any of these experiments of concern:

No	Yes	
☒	☐	Demonstrate how to render a vaccine ineffective
☒	☐	Confer resistance to therapeutically useful antibiotics or antiviral agents
☒	☐	Enhance the virulence of a pathogen or render a nonpathogen virulent
☒	☐	Increase transmissibility of a pathogen
☒	☐	Alter the host range of a pathogen
☒	☐	Enable evasion of diagnostic/detection modalities
☒	☐	Enable the weaponization of a biological agent or toxin
☒	☐	Any other potentially harmful combination of experiments and agents
